# Gynecologic imaging errors and pitfalls: how to avoid misdiagnosis on pelvic MRI

**DOI:** 10.1186/s13244-026-02378-2

**Published:** 2026-09-17

**Authors:** Alexandre Faure, Edwige Pottier, Marie Florin, Léo Razakamanantsoa, Pascal Rousset, Isabelle Thomassin-Naggara

**Affiliations:** 1 Assistance Publique Hopitaux de Paris (AP-HP), Radiology Imaging and Interventional Radiology Department, Tenon Hospital, Sorbonne University, Paris, France; 2 Laboratoire HEKA, INRIA & INSERM, Paris Cité University, PariSanté Campus, Paris, France; 3 Private Hospital NCT+, Tours, France; 4 Saint-Antoine Research Center (CRSA), INSERM UMR-S 938, CNRS, Sorbonne University, Paris, France; 5 Service d’imagerie médicale et interventionnelle, Hospices Civils de Lyon, Pierre Bénite, France; 6 Lyon Sud University Hospital, Lyon 1 Claude Bernard University, EMR 3738, Pierre Bénite, France

**Keywords:** Gynecology, MRI, Endometriosis, O‑RADS MRI, FIGO 2023

## Abstract

**Abstract:**

Pelvic magnetic resonance imaging (MRI) plays a pivotal role in evaluating gynecologic diseases, offering superior soft-tissue characterization, multiplanar imaging, and functional assessment compared to ultrasound and computed tomography. This article proposes evidence-based, standardized MRI protocols and a reproducible interpretative framework to enhance diagnostic accuracy, reduce misdiagnoses, and support multidisciplinary management in routine clinical practice. Key insights cover MR imaging of major gynecologic diseases: endometriosis, adnexal masses including the O-RADS MR scoring system, and pathological hypothesis as well as uterine diseases including endometrial, cervical, and myometrial benign and malignant conditions. For all situations, we present typical and atypical presentations as well as mimickers. This article will give some tips and tricks to help the radiologist in clinical routine for detecting and differentiating the main issues that impact patient management. By adopting standardized protocols and reports with adequate pathology knowledge, radiologists can improve patient outcomes through precise staging, surgical planning, and personalized care.

**Key Points:**

- **Question** How can radiologists avoid common diagnostic errors and pitfalls when interpreting pelvic MRI for gynecologic diseases, including endometriosis, uterine tumors, and adnexal masses?
- **Findings** Knowledge of common pitfalls, standardized MRI protocols with Dixon-based T1-weighted sequences, diffusion-weighted imaging, and dynamic contrast enhancement, if necessary, combined with structured reporting frameworks including O-RADS MRI and FIGO 2023, reduces misdiagnosis across gynecologic conditions.
- **Critical Relevance Statement** This article critically reviews typical and atypical presentations alongside common mimickers of major gynecologic diseases on pelvic MRI, providing radiologists with practical evidence-based strategies to improve diagnostic accuracy, reduce unnecessary interventions, and support multidisciplinary clinical decision-making.

**Graphical Abstract:**

**Figure:**
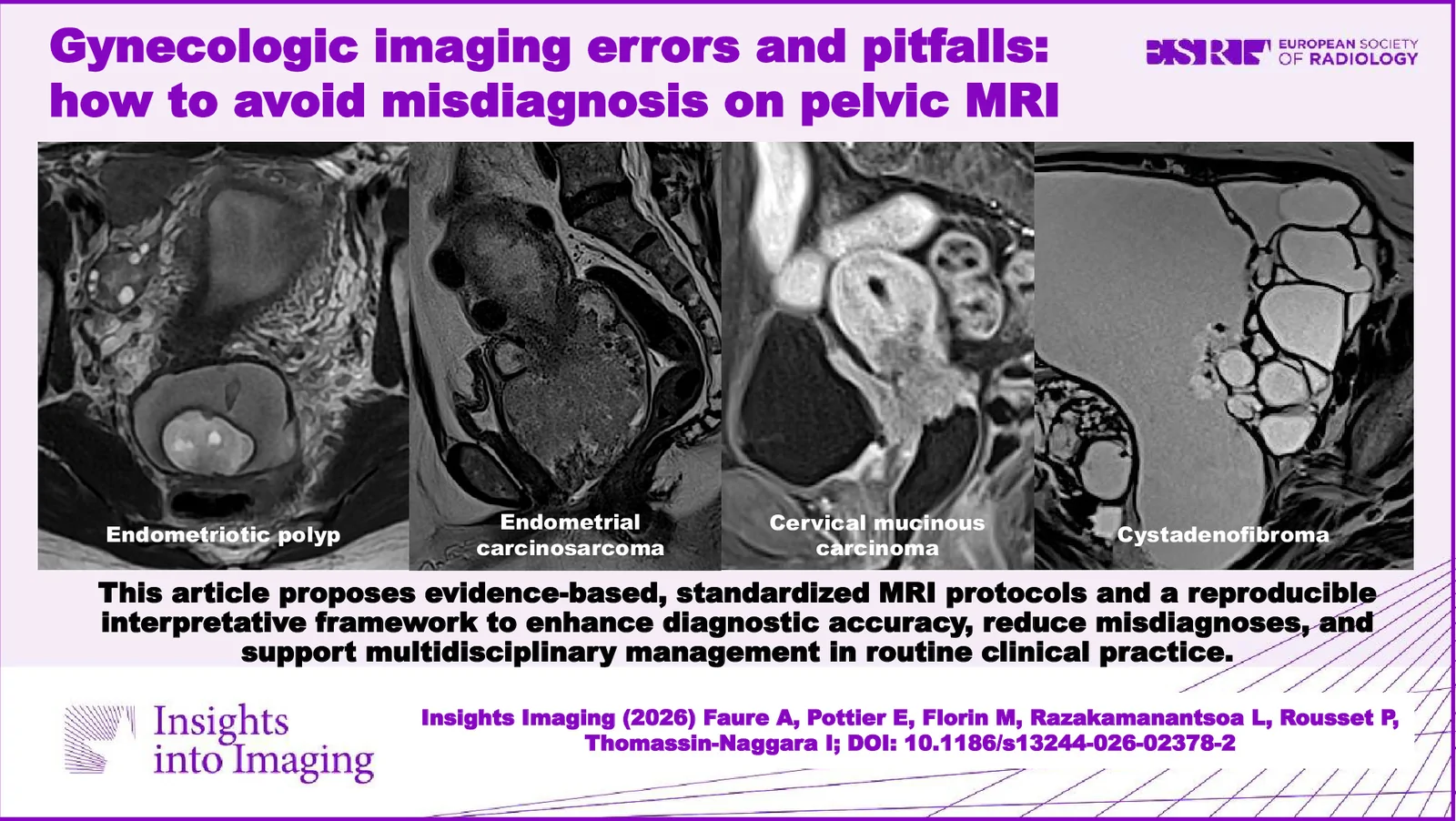

## Introduction

Pelvic MRI has become a cornerstone in the evaluation of gynecologic diseases, providing superior soft-tissue contrast, multiplanar capability, and functional information that far exceeds those of ultrasound and CT in many clinical scenarios [1, 2]. Beyond simple lesion detection, MRI is now expected in both benign gynecological diseases such as the characterization of adnexal masses [3, 4], the mapping of deep infiltrating endometriosis [5, 6], or the discrimination benign uterine leiomyomas from atypical variants and malignant mimickers [7, 8], and malignant gynecological tumors with the staging of ovarian and uterine malignancies (endometrium, cervix, vulva and vagina) [9–12].

In this context, non-standardized protocols or inconsistent reporting can substantially impact diagnostic performance, multidisciplinary decision-making, and ultimately patient care. A robust core pelvic MRI protocol—possibly incorporating patient preparation, mandatory T2 and T1-weighted (T1W) planes (optimally performed Dixon techniques) and functional sequences including diffusion-weighted imaging and dynamic contrast-enhanced sequence is crucial for liquid and solid component characterization. An optimal complete MR protocol is crucial as tissue characterization constitutes the primary added value of MRI over transvaginal ultrasonography. Equally important is a structured reading strategy that proceeds from lesion origin to internal architecture and finally formal risk categorization, while remaining vigilant for key pitfalls and mimickers.

The aim of this article is to propose practical, evidence-based MRI protocols for pelvic imaging and to detail a reproducible interpretative framework by emphasizing typical and atypical presentations, as well as common mimickers across the main gynecological diseases. We seek to help radiologists reduce misdiagnoses and better support clinical and surgical management in routine clinical practice.

## Core pelvic MRI protocol

Patient preparation should include abdominal compression, administration of an antiperistaltic agent, a short fasting period (> 3 h), and maintenance of a moderately filled bladder to improve uterine and adnexal delineation.

A female pelvic MR protocol can be performed equally on 1.5-T and 3-T MRI. The protocol must always include a multiplanar T2-weighted (T2W) acquisition, 3D or at least two 2D planes (one plane strictly perpendicular and one parallel to the long axis of the uterus, preferably with high-resolution 2D sequences, to optimize assessment of the junctional zone, cervical stroma, and parametria). Vaginal gel should be considered in the context of cervical pathology and endometriosis to increase delineation of the vagina [13]. At least one T1W sequence with different contrast is required to identify the different cystic components. In this setting, the use of a Dixon-based acquisition is mandatory to reliably differentiate fat, blood products, and protein-rich fluid, ensuring accurate characterization of adnexal and pelvic lesions. In addition, a systematic evaluation of the retroperitoneal lumbar space and kidneys is required, and dedicated T2W and DW lumbopelvic sequences should be acquired to assess para-aortic and lumbar lymph nodes and document the position of the left renal vein, which may influence pelvic surgery. A slice thickness of 5 mm is enough for this large coverage. Gadolinium use is mandatory when tissue characterization and/or local extension is needed (Table 1). The typical slice thickness range should be less than or equal to 3 mm for pelvis sequences in order to detect papillary projections or small recurrences for cancer, for example. For all indications, a coronal T2 short echo sequence is necessary to detect ureterohydronephrosis.

**Table 1:** MR protocol

	Pelvic 2D Sag T2W	Pelvic thin slices 2D axial T2W or 3DT2W	Lumbopelvic 2DT2 axial or coronal HASTE	DW sequence	High spatial 3DT1W with FS Before and after gadolinium
Endometriosis	Pelvic	3D > 2D If 2D: oblique USL	Coronal HASTE	Only if atypical adnexal mass Axial pelvic	Before only except if: atypical adnexal mass, wall nodule, somatic nerves
Adnexal mass	Pelvic	+++ 2D = 3D	Coronal HASTE	Axial pelvic	Always both except if significant fatty content before injection Always includes DCE and subtraction
Adenomyosis	Pelvic	3D > 2D	Coronal HASTE	-	-
Atypical myometrial tumors	Pelvic	3D	Coronal HASTE	Axial pelvic	Unenhanced T1W only, if ADC > 1.23 × 10⁻³ mm²/s anywhere in the tumor Subtraction +++
Endometrial cancer staging	Pelvic	3D > 2D If 2D: oblique uterine corpus	Axial 2DT2W	Lumbopelvic axial Sagittal HR pelvic	Always At 2min after gadolinium injection ± DCE if premenopausal
Cervical cancer staging	Pelvic	3D > 2D If 2D: oblique USL	Axial 2DT2W	Lumbopelvic axial Sagittal HR pelvic	Always Includes DCE

*USL* uterosacral ligament

## Endometriosis

### Typical presentation

Endometriosis classically presents with ectopic endometrial glands and stroma that generate cyclic hemorrhage, fibrosis, and chronic inflammation. Typical manifestations include ovarian endometriomas, usually showing T1W high signal with T2W shading, and deep infiltrating endometriosis affecting the uterosacral ligaments, torus uterinum, rectovaginal septum, and bowel [6, 14]. These lesions commonly appear as fibromuscular plaques of low T2W signal intensity with spiculated or nodular margins, often associated with surrounding tethering and distortion of pelvic compartments. Superficial peritoneal implants may be subtle, occasionally seen as small foci of T1 hyperintensity or fibrotic puckering. Disease involving the bladder or ureters follows similar patterns, with T2-dark nodules or plaques sometimes accompanied by mural thickening or hydroureteronephrosis.

The description of deep pelvic endometriosis is optimally performed using compartmental analysis to standardize endometriosis reports, help surgical planning and multidisciplinary decision-making, and optimize communication [6, 15, 16]. Two main classifications were developed and externally validated with MRI: the ENZIAN scoring system and the dPEI scoring system. ENZIAN classification mostly stages lesions based on size and thus has shown a poor reproducibility for uterosacral ligament (USL) and parametria description [6]. The dPEI scoring system is mainly weighted based on the presence of lateral locations, which is the main source of complexity for surgery, and may help to improve the selection of women who would be referred to expert surgical centers.

### Atypical presentations

**Fig. 1:**
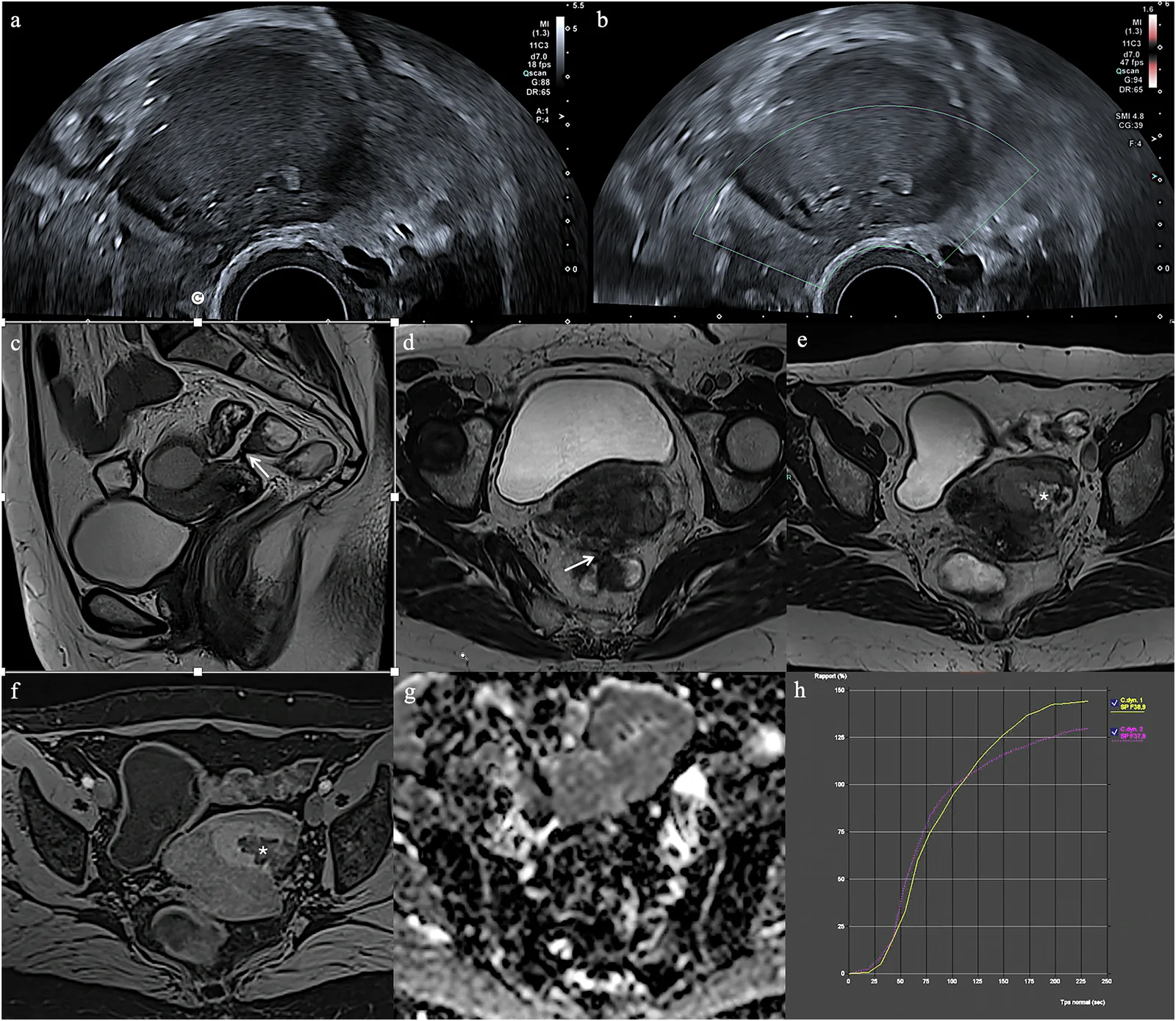
Endometrioid cystadenocarcinoma in the context of deep pelvic endometriosis. Ultrasonography and MRI. **a**, **b** Ultrasonography with power Doppler; **c** sagittal T2-weighted sequence; **d**, **e** axial T2-weighted sequence; **f** axial T1 water-only image from a T1-weighted Dixon sequence; **g** ADC map; **h** TIC from DCE MR sequence (lesion in purple and external myometrium in yellow). Ultrasonography shows an adnexal mass with echogenic cystic content and a solid part without evident vascularization. MRI shows a deep pelvic endometriosis on mediocentral and posterocentral compartments (white arrows) associated with an ovarian cyst with endometriotic liquid that contains a group of papillary projections (*). This solid tissue is high T2W signal with diffusion restriction and enhances according to a high-risk time-intensity curve (purple curve). Thus, this mass was scored O-RADS MR 5 and corresponded to endometrioid cystadenocarcinoma

**Fig. 2:**
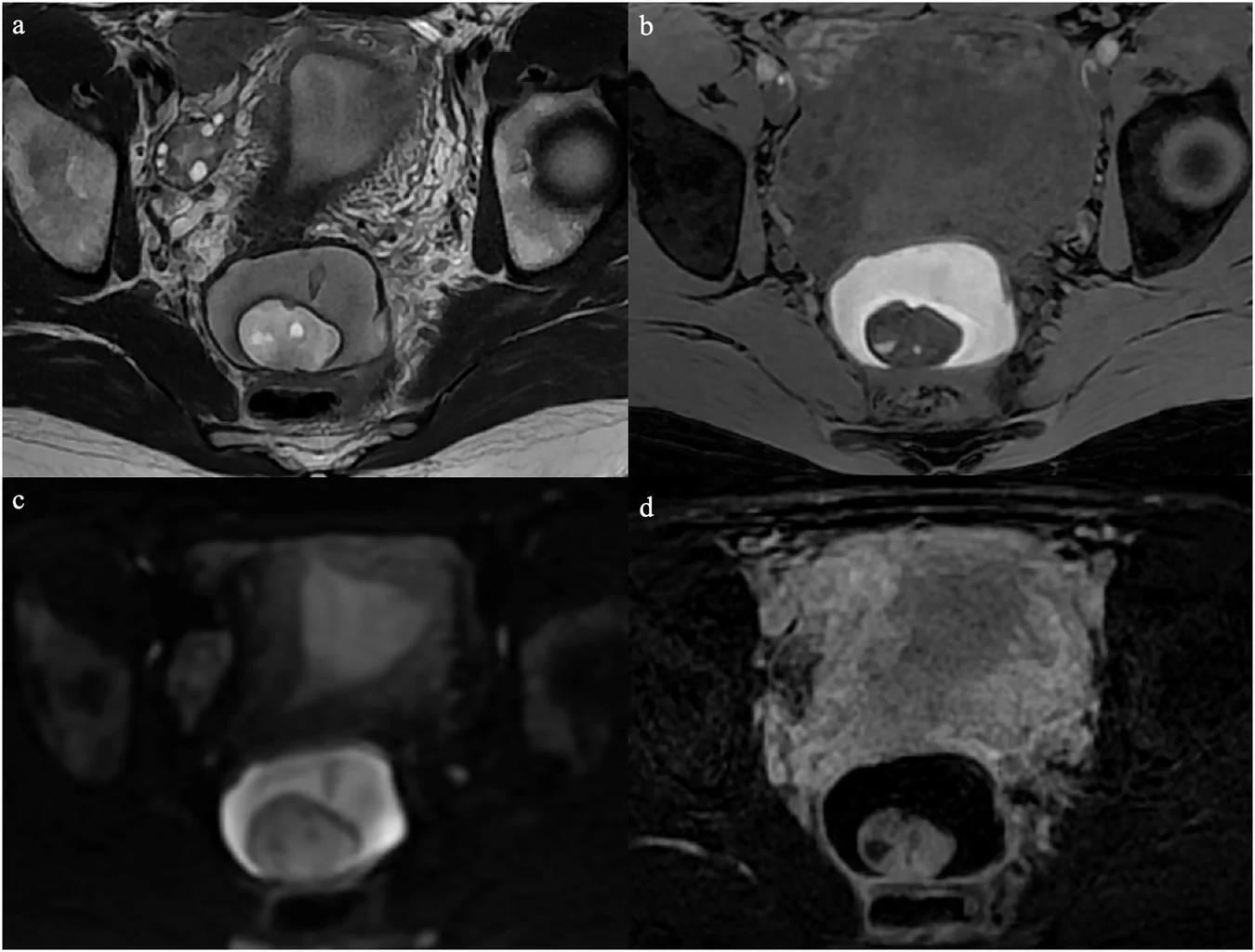
Endometriotic polyp. **a** Axial T2-weighted sequence; **b** axial T1 water-only image from a T1-weighted Dixon sequence; **c** DWI sequence; **d** postcontrast fat-saturated T1-weighted sequence. The images show a posterior pelvic cyst with endometriotic liquid that contains a solid component with high T2W signal and low DWI signal. Injection shows enhancement of the solid part. TIC from DCE MR sequence (not presented here) shows an intermediate-risk time-intensity curve. Thus, the lesion was classified as O-RADS MR score 4. This case highlights the usefulness of subtracted images

Beyond these classical features, ovarian endometriosis may display atypical presentations. The main issue is to recognize malignant transformation (1–2% of endometriomas) from other diagnoses when a tissue component is identified in an endometriotic cyst (Fig. 1). Typically, solid tissue in malignant degeneration will be scored O-RADS MR 4 or 5 and display a partial loss of T1 hyperintensity of the cystic part [17]. Endometrioid and clear cell carcinomas are the most common histologic subtypes [18]. In the differential diagnosis, endometriotic polyps arising within or adjacent to endometriotic tissue may also present as intracystic soft-tissue nodules, requiring careful assessment of enhancement and internal architecture (Fig. 2). Acute or subacute inflammation may alter the usual imaging appearance of endometrioma, leading to increased wall thickening, perilesional edema, or more pronounced enhancement, potentially mimicking infection or neoplastic transformation (Fig. S1). In pregnant patients, decidualized endometriomas may also present with rapidly growing, highly vascular mural nodules, sometimes mimicking malignant transformation. Unlike carcinoma, these nodules often show high T2 signal, smooth regular contours, and relatively high ADC values, and typically regress on follow-up after delivery. Correlation with gestational age and interval imaging is therefore crucial to avoid unnecessary surgery (Fig. S2).

To summarize, in the presence of an enhanced mural nodule and a loss of high T1W signal, malignancy should be considered [19]. The absence of internal enhancement serves as the main clue to benign intracystic coagulum, possibly mimicking a nodule.

Polypoid endometriosis is a rare variant of endometriosis that may clinically, surgically, and grossly resemble a neoplastic lesion. This benign form of endometriosis typically mimics malignant tumors due to its infiltrative tissue pattern, often resembling DIE with features suggestive of malignant transformation [20]. Preoperative diagnosis can be challenging, particularly when the condition presents in atypical locations, in the absence of a previous history of endometriosis, or without other typical endometriotic MRI signs, as demonstrated in our case (Fig. 2). However, several imaging features can aid in narrowing the differential diagnosis [21]. These include positive signs, such as intermediate signal intensity on T2-W, microcystic hemorrhagic spots, and a fibrous surrounding rim around the nodular component. Additionally, negative functional signs of malignancy are crucial in differentiating polypoid endometriosis from malignant tumors. These include the absence of diffusion restriction on diffusion-weighted imaging (DWI) and the lack of pronounced tumoral enhancement on contrast-enhanced sequences. Furthermore, the absence of ancillary signs such as lymphadenopathy or peritoneal metastases further supports the benign nature of the lesion and helps exclude malignant transformation of the endometriotic lesion or other malignancies in the differential diagnosis. Ultimately, the definitive diagnosis of polypoid endometriosis requires histopathological examination.

### Mimickers

Several conditions can mimic endometriosis and must be recognized to avoid overdiagnosis. A meticulous MRI analysis and careful consideration of a broad spectrum of differential diagnoses, including both pathologic and non-pathologic causes, are essential.

The first category of mimickers arises from T1-hyperintense findings on fat-suppressed T1-weighted (T1FSW) images [22], which may resemble cystic endometriotic lesions across the three phenotypes of endometriosis in the different pelvic compartments. Sources of false positives in this category primarily include non-endometriotic hemorrhage. The most common mimickers of endometrioma are functional hemorrhagic cysts: Endometriomas typically show uniformly high T1 signal, persistent hyperintensity on T1FSW images, and at least partial T2 ‘shading,’ often associated with the T2 dark spot sign, which has high specificity for chronic blood products. In contrast, hemorrhagic cysts are usually transient, unilocular, and may present a variable T2 signal with or without shading, but they tend to resolve or significantly decrease in size on short-interval follow-up imaging and lack the combination of persistent T1 hyperintensity, dark spots, and chronicity that characterizes endometriomas. Radiologists should therefore integrate morphology, signal pattern, and evolution over time to avoid overcalling functional hemorrhagic cysts as endometriosis [14, 23]. Other causes of misdiagnosis include high protein content (as seen in cysts of the vagina, vulva, urachus, or retroperitoneal/peritoneal cysts) (Fig. S3), melanin, fecal matter (within the appendix or sigmoid diverticula) (Fig. S4), or MRI-related artifacts (such as vascular flow-related enhancement or calcification) (Fig. S5).

**Fig. 3:**
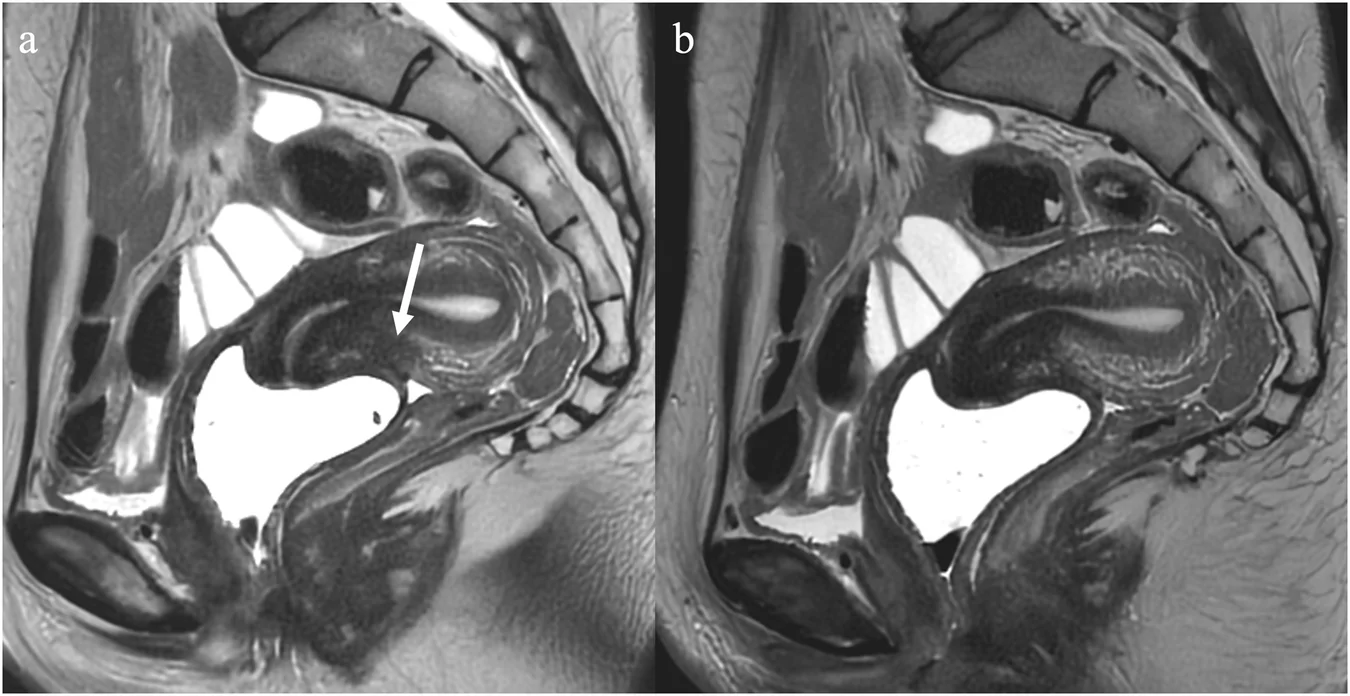
Uterine contraction in a 21-year-old woman with chronic catamenial pelvic pain. **a**, **b** Sagittal T2-weighted sequences at two different times. The initial MR images show a T2-hypointense focal thickening (arrow) of the torus uterinum. The second image shows the absence of abnormality of the torus corresponding to complete resolution of uterine contraction

The second category of false-positive findings consists of T2-hypointense thickenings or pseudo-lesions on T2-W images, which may simulate deep infiltrating endometriosis (DIE) [24]. These include anatomical variants (Fig. 3), fibrous connective tissue, infectious conditions (Fig. S6), benign or malignant tumors, fecal material, surgical devices, and post-surgical fibrotic scars (Fig. S7). Such entities may present as hypointense thickenings, nodules, or infiltrative tissue-like masses involving pelvic organs, potentially leading to distortion of normal anatomy and loss of normal physiological signal, particularly within the muscularis layer of affected structures. The use of gadolinium-based contrast agents may be helpful in selected cases to refine the differential diagnosis.

## Uterine disease

### Adenomyosis

The diagnosis of adenomyosis based solely on the thickening of the junctional zone is a source of misdiagnosis, as this indirect sign is nonspecific. The presence of cystic or hemorrhagic lesions of 2–7 mm in the superficial myometrium is more specific but lacks sensitivity [25] (Fig. S8a, b). Furthermore, the secondary myometrial smooth-muscle hyperplasia and hypertrophy that characteristically accompany adenomyotic foci can closely simulate true focal masses, further compounding diagnostic confusion [25].

### Atypical presentations

Focal adenomyosis represents a localized, mass-like form of adenomyosis in which ectopic endometrial glands and stroma cluster within a limited myometrial area rather than diffusely involving the junctional zone. On MRI, it usually appears as an ill-defined intramyometrial region of low T2 signal, often contiguous with a thickened junctional zone and containing punctate T2/T1 hyperintense foci corresponding to hemorrhagic glands (Fig. S8c, d). Compared with a leiomyoma, focal adenomyosis tends to have blurrier margins, less mass effect, and lacks a peripheral low-signal capsule, which helps avoid misclassification.

### Mimickers

**Fig. 4:**
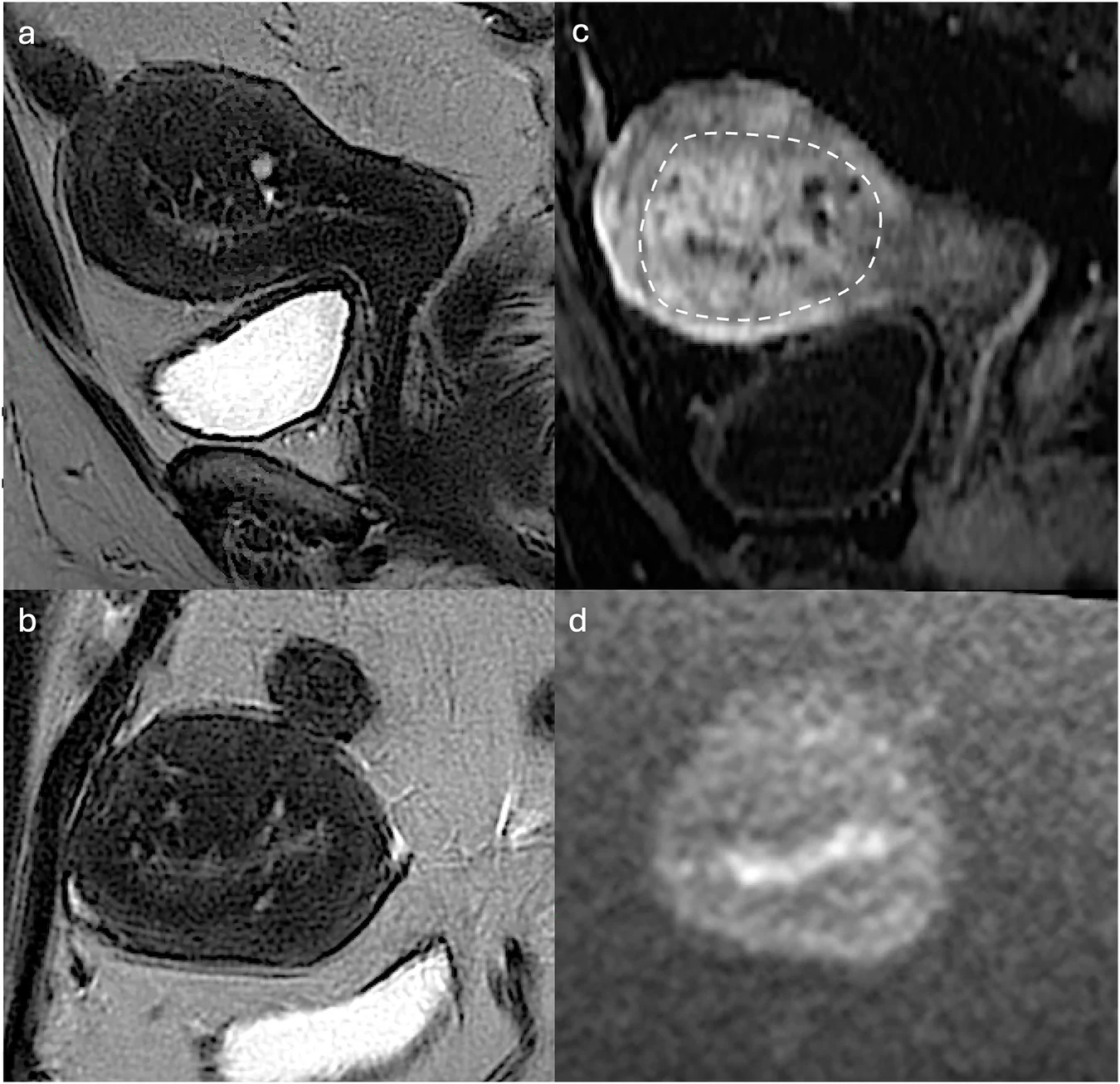
Adenomyosis mimicker: a case of endometrial stromal sarcoma (ESS). **a** Sagittal T2-weighted sequence; **b** sagittal postcontrast fat-saturated T1-weighted sequence; **c** coronal T2-weighted sequence; **d** coronal DWI sequence. The images show an ill-defined lesion extending into both the endometrial cavity and the uterine wall, with a cystic component. The anterior uterine wall is difficult to distinguish from the lesion, with possible residual normal low-T2 striations and small cysts possibly mimicking adenomyosis. Postcontrast sequence confirms the ill-defined nature (white broken line) of the lesion with high diffusion signal. ESS, endometrial stromal sarcoma

Endometrial stromal sarcoma (ESS) is an important mimicker of uterine adenomyosis, particularly in atypical or rapidly growing masses. On MRI, ESS often presents as an infiltrative myometrial lesion with ill-defined margins, replacing or expanding the myometrium rather than forming a well-circumscribed mass [26]. It commonly shows high T2 signal, restricted diffusion, and avid, early enhancement, reflecting its cellularity. DWI signal is therefore particularly useful in this setting [27] (Fig. 4). Intramyometrial “worm-like” nodules or bands of low T2 signal may correspond to lymphovascular invasion, a characteristic but not universal feature. Unlike adenomyosis, ESS, which frequently also distorts the junctional zone, may extend into the endometrium or parametrium. Because its presentation can also overlap with degenerated or cellular leiomyomas, ESS should be suspected when a uterine mass shows aggressive growth, irregular borders, marked diffusion restriction, or atypical enhancement, particularly in peri- or postmenopausal women.

### Leiomyomas

Benign uterine leiomyomas typically demonstrate a well-circumscribed, round or lobulated morphology with sharp borders. The most common form, hyaline leiomyoma, accounts for the majority of cases and displays a well-circumscribed morphology, marked homogeneous low T2W signal, and low T1W signal [28]. Diffusion signal and ADC should not be evaluated as they can be falsely suggestive of malignancy. This is because dense bundles of fusiform smooth muscle cells within the extracellular matrix limit Brownian motion of water molecules, yielding low ADC values (typically < 1.3 × 10⁻³ mm²/s) [29]. These typical features allow a reliable diagnosis in most patients.

### Atypical presentations

However, several atypical or degenerated variants may alter the imaging appearance and occasionally mimic malignant disease. Non-enhancing patterns include cystic degeneration, fatty degeneration (lipoleiomyoma), and coagulative necrosis. These variants manifest as regions of high T2W signal intensity, show T1W hyperintensity when containing blood products or fat, demonstrate an absolute absence of internal enhancement, and exhibit high diffusion signal with correspondingly elevated ADC values. Cystic degeneration, in particular, can create a multilocular or fluid–fluid level appearance that may be mistaken for an adnexal lesion.

**Fig. 5:**
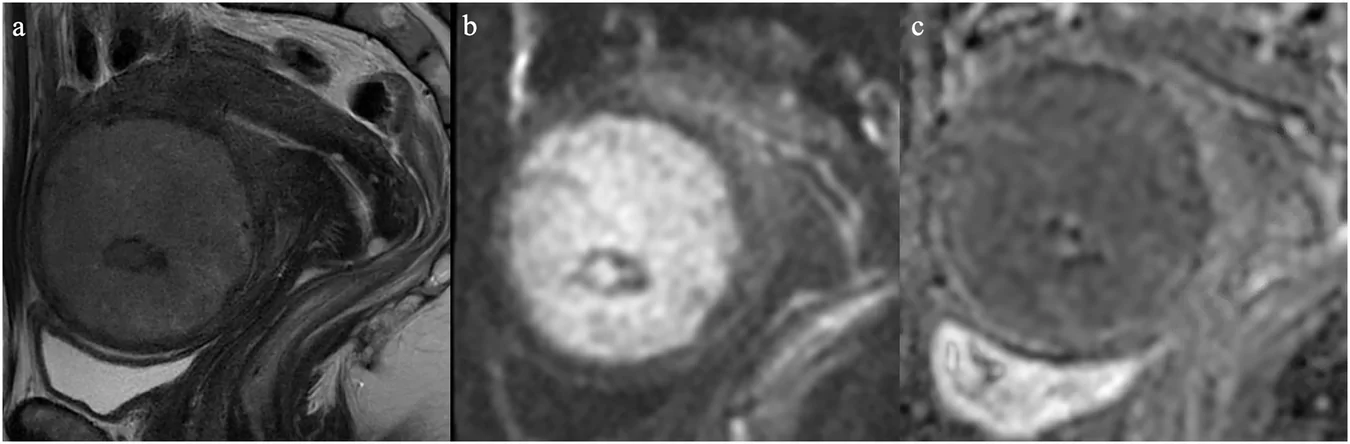
Cellular leiomyoma in a premenopausal woman. **a** T2-weighted sequence; **b** DWI sequence; **c** ADC MAP. The images show a myometrial lesion with intermediate T2W signal, high signal intensity, and an ADC map showing low values, measured at 0.9 × 10⁻³ mm²/s. The lesion showed homogeneous enhancement and smooth margins, allowing classification as score 3 according to Zlotykamien-Taieb et al [7]

Among the enhancing atypical forms, edematous leiomyomas exhibit very high T2 signal with streaky or heterogeneous enhancement, while cellular leiomyomas demonstrate intermediate T2 signal, marked diffusion hyperintensity with low ADC (potentially mimicking sarcoma) (Fig. 5), and a homogeneous and avid early enhancement; despite this aggressive profile, they often remain small in size, well-circumscribed with preserved borders [30]. Fumarate hydratase (FH)–deficient leiomyomas may present with intermediate T2W signal with high T2W microcystic degeneration, high diffusion restriction, intense enhancement, regular borders, requiring correlation with patient age and syndromic context (Fig. S9) [31]. Myxoid leiomyomas appear markedly T2-hyperintense and pseudocystic, with preserved low enhancement. Finally, bizarre (atypical) leiomyomas may contain irregular internal architecture, variable T2 signal, and moderate enhancement, yet crucially maintain non-infiltrative borders.

**Fig. 6:**
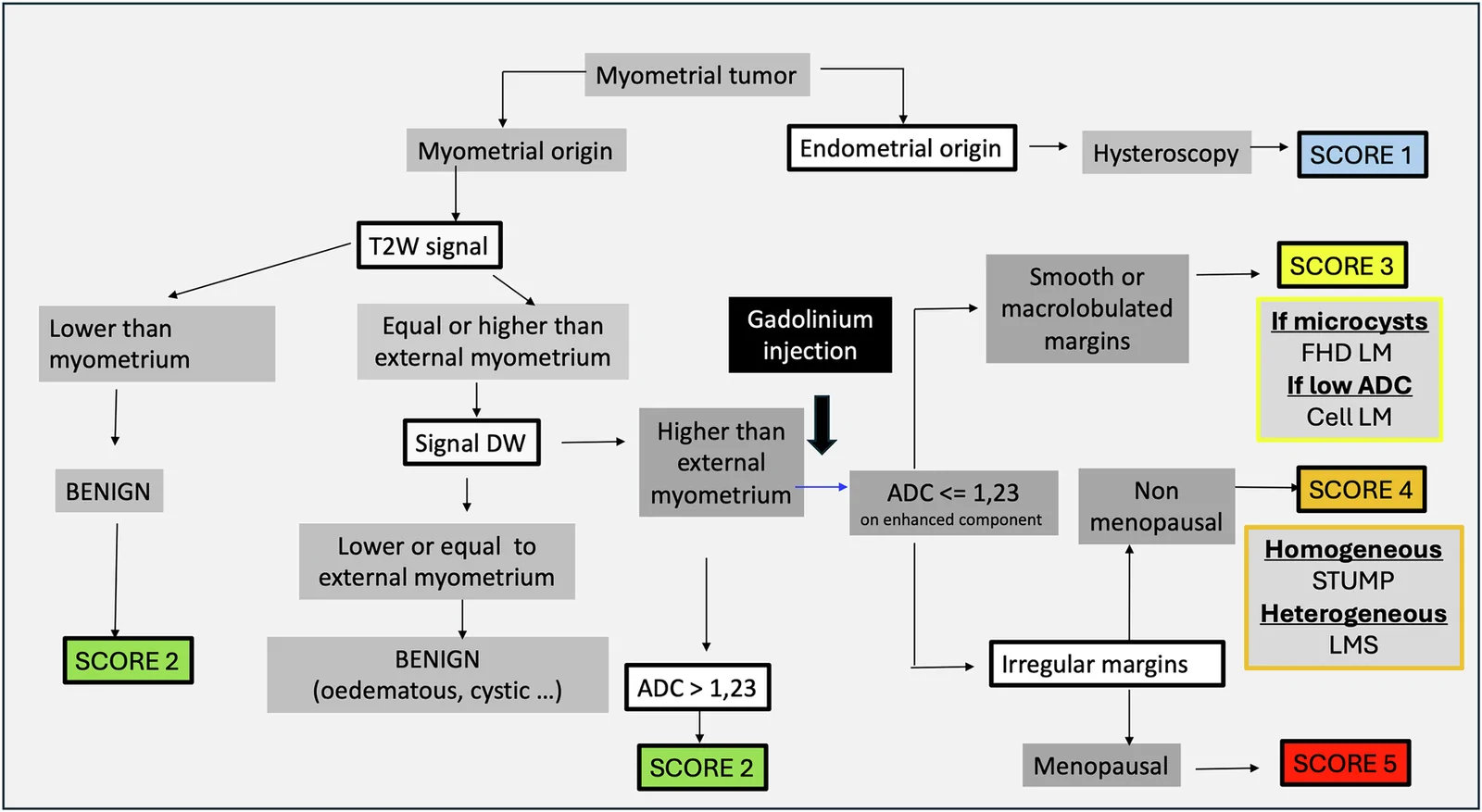
Myometrial lesions scoring system adapted from Zlotykamien-Taieb et al [7]

Across all variants, the key imaging discriminator remains margin morphology [7]: benign leiomyomas, regardless of their degeneration pattern, almost always preserve smooth, well-defined contours. Integrating T2 appearance, diffusion behavior, ADC values, and lesion margins in a scoring system model recently published [7] (Fig. 6), allows reliable prediction of malignancy and accurately differentiates typical fibroids, degenerated atypical variants, and potential malignant mimickers. Table 2 synthesizes the key morphological and functional features of typical leiomyomas, their benign variants, and malignancies.

**Table 2:** Leiomyoma, variants, and malignant form

Type	Morphology	T2 signal	T1 signal	DWI	ADC value	Enhancement
Typical = Hyalin	Well-circumscribed, round	Low	Low	Low	Not evaluated	Low homogeneous
Cystic	Well-circumscribed, round	High	Low	Low	> 2	None
Edematous	Well-circumscribed, round	High	Low	Low	> 1.2	High homogeneous
Myxoid	Well-circumscribed, round	High	Low	Low	> 2	Low
Lipoleiomyoma	Well-circumscribed, round	High	High (fat)	Low	Not evaluated	Heterogeneous (fat saturation)
Cell-LM	Well-circumscribed, round, or irregular borders	Intermediate	Low	High	< 1.1	High homogeneous
FHD-LM	Well-circumscribed, round	Heterogeneous	Areas of high signal (blood)	High	< 1.1	High heterogeneous
STUMP	Well-circumscribed, irregular border	Heterogeneous	Areas of high signal (blood)	High	< 1.1	High heterogeneous
Leiomyosarcoma	Ill-defined margins, infiltrative behavior	Intermediate	Areas of high signal (blood)	High	< 1.1	High heterogeneous

*DWI* diffusion-weighted imaging, *FHD-LM* fumarate hydratase deficient, *Cell-LM* cellular leiomyoma

### Mimickers

Several conditions may mimic uterine leiomyomas on MRI and must be considered in the differential diagnosis. Accessory and cavitated uterine mass (ACUM) and adenomyoma can appear as well-circumscribed masses with variable T2 signal and areas with high T1 signal, mimicking atypical or degenerated fibroids [32]. In reproductive-age patients, interstitial pregnancy may mimic a myometrial mass on initial imaging, making serum β-hCG testing mandatory whenever pregnancy is clinically possible. Focal adenomyosis and adenomyomas are also important mimickers of leiomyomas as described above. Recognizing these features helps avoid misclassifying adenomyotic lesions as atypical or degenerated fibroids, especially in symptomatic reproductive-age women (Fig. S10).

Mostly encountered in menopausal women, STUMP and leiomyosarcomas always present an intermediate or high T2 and DW signal and mostly ill-defined margins [33] (Figs. S11 and 7). Most of them will have a low ADC value [28]. An ADC value of 0.9 × 10⁻³ mm²/s has been previously suggested by different studies, showing an accuracy ranging between 88 and 95% in order to restrict unnecessary hysterectomy [33, 34]. However, in light of recent work, ADC criteria should not be considered alone and should be combined with other features [7]. In this setting, an ADC > 1.23 × 10⁻³ mm²/s has been proposed for its very high negative predictive value (NPV) to exclude malignancies with the same diagnostic value as low T2W Signal and low DW signal compared to external myometrium (i.e NPV = 100%). The analysis of margins and the hormonal status of the patient must also be considered. The only exception could be myxoid subtypes of sarcoma, which may demonstrate high ADC values, but can be recognized by their characteristic high T2W signal. In postmenopausal women, any symptomatic or enlarging “leiomyoma”, even without internal enhancement (completely necrotic), should also be presumed malignant until proven otherwise.

**Fig. 7:**
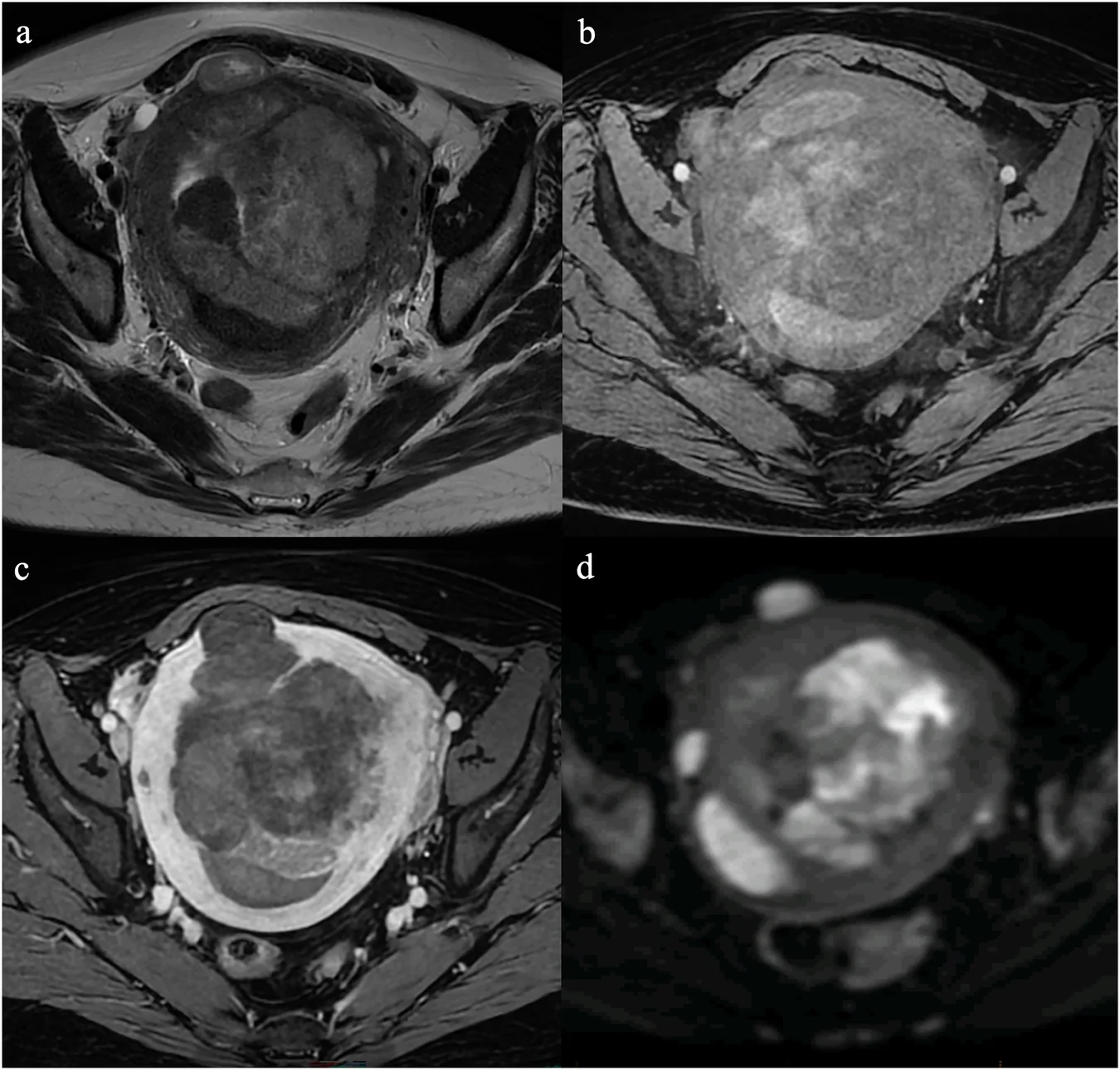
Leiomyosarcoma. **a** Axial T2-weighted sequence; **b** axial water-only image from a T1-weighted Dixon sequence; **c** axial postcontrast fat-saturated T1-weighted sequence; **d** axial DWI sequence. Image is showing a heterogenous ill-defined myometrial lesion with intermediate T2W signal, spontaneous T1W signal corresponding to hemorrhage. Postcontrast imaging demonstrates large necrotic areas and infiltrative peripheral parts with relatively high DWI signal. ADC map (not shown) demonstrated values as low as 0.8 × 10⁻³ mm²/s in the enhancing component. As the patient was postmenopausal, the lesion was classified as score 5 [7]

## Endometrial malignancies

The updated 2023 FIGO system has made endometrial cancer classification more prognostic, but also more complex for applying pre-therapeutic FIGO staging on MRI. Tumors must now be staged by integrating traditional anatomic factors (depth of myometrial invasion, cervical and adnexal extension, nodal status) with histologic type and molecular subgroup (POLE-mutated, MMR-deficient, p53-abnormal, NSMP) [35]. In practice, this is challenging because biopsy samples may not fully represent the whole tumor. Intratumoral heterogeneity is common, and molecular testing is not yet universally available. For radiologists, MRI still primarily depicts anatomy, so translating these anatomical features to pathology and biologically driven categories requires the correct application of the new stage boundaries. It requires revised protocols, structured reporting, and close collaboration with pathology and oncology teams, as emphasized in the recently updated ESUR guidelines [9]. Following this, we highlight some important features.

FIGO stage IA3 designates low-grade endometrioid endometrial carcinoma confined to the uterus with simultaneous low-grade endometrioid involvement of a single ovary, provided stringent criteria are fulfilled (Fig. S12a, b). According to the 2023 FIGO revision, these cases must show no more than superficial myometrial invasion (< 50%), no substantial lymphovascular space invasion (LVSI), no additional metastatic disease, and unilateral ovarian involvement limited to the ovary without capsular invasion or rupture (pT1a-equivalent). When any of these conditions is not met (bilateral ovarian disease, deep myometrial invasion, extensive LVSI, extra-ovarian spread, or capsular breach), the adnexal disease is interpreted as extensive spread of endometrial carcinoma and reclassified as FIGO stage IIIA1.

Anterior vaginal wall metastases often show a characteristic horseshoe configuration on MRI, with tumor tissue extending into the lateral and anterior paravaginal spaces, then curving around the urethra (Fig. S12c, d). Spread follows the paths of least resistance laterally and anteriorly, while the fibrous tissue between the urethra and vagina acts as a non-distensible barrier, redirecting tumor growth around rather than through this plane. On T2W and postcontrast images, this results in a semicircular or horseshoe-shaped soft-tissue cuff hugging the anterior vaginal wall and peri-urethral region, a pattern that should prompt careful assessment of urethral and bladder neck involvement [36].

### Atypical imaging patterns and unusual presentations

One possible atypical presentation is polypoid endometrioid adenocarcinoma, which is an endometrioid-type endometrial carcinoma that grows predominantly as a polypoid mass projecting into the uterine cavity, often resembling a benign endometrial polyp on imaging and hysteroscopy. On MRI, it usually appears as an intracavitary, lobulated soft-tissue lesion arising from the endometrium, with intermediate T2 signal, and true diffusion restriction, in contrast to benign polyps that show microcysts and no ADC restriction. While an ADC < 0.8 × 10⁻³ mm²/s is suggestive of endometrial cancer [37], an endometrial cancer cannot be excluded on ADC values and a hysteroscopy must be indicated. Myometrial invasion may be focal and sometimes difficult to assess when the polyp is large and distends the cavity. Because this pattern can be deceptively “polyp-like,” careful evaluation of diffusion and junctional zone integrity is essential to avoid underestimating malignancy.

**Fig. 8:**
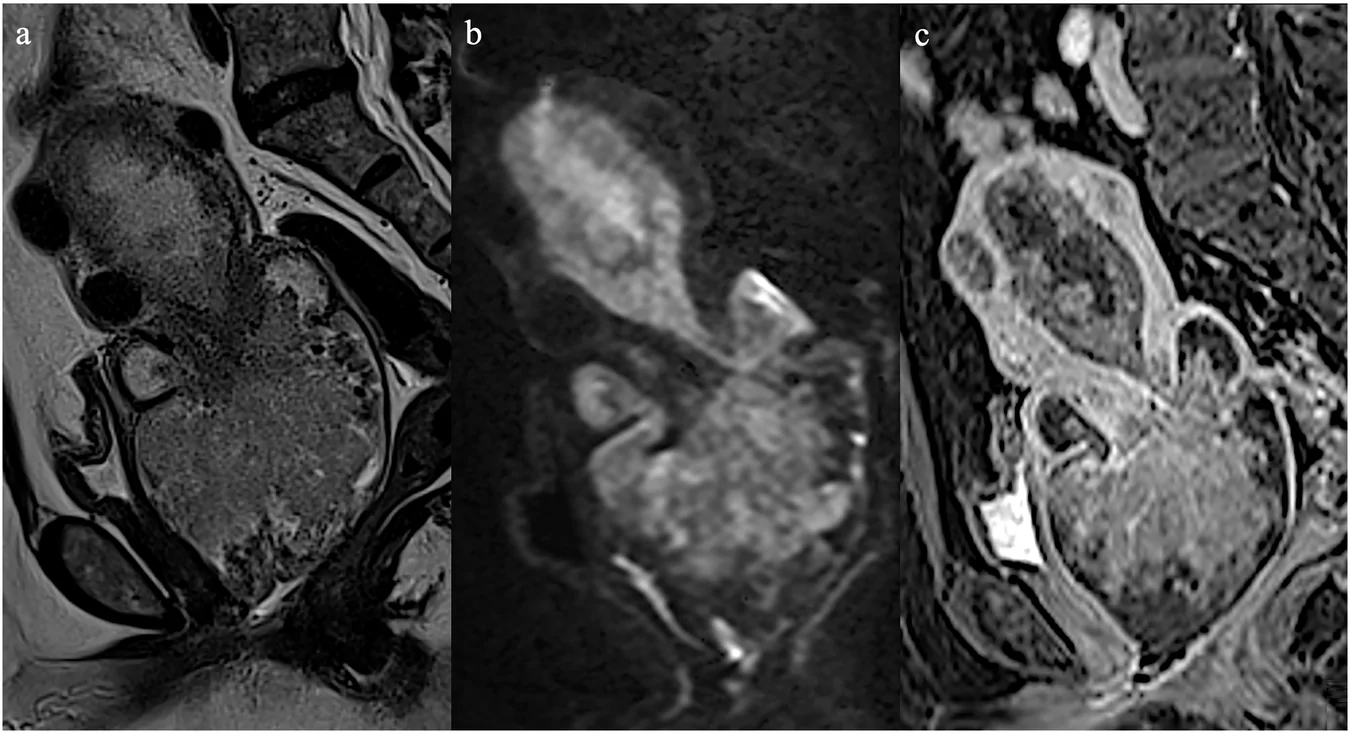
Endometrial carcinosarcoma. **a** Sagittal T2-weighted sequence; **b** sagittal DW sequence; **c** postcontrast fat-saturated T1-weighted sequences. High-grade endometrial carcinosarcoma distending the endometrial cavity with typical extension to the cervix, which is extended without true stromal invasion

Carcinosarcoma is another mimicker. These tumors often fill and distend the uterine cavity, protruding into the cervix [38]. On MRI, they typically demonstrate an intermediate T2W signal intensity interspersed with distinct flow voids. DWI demonstrates marked diffusion restriction within the viable solid portions, and enhancement after gadolinium is irregular [39, 40]. Compared with low-grade endometrioid carcinomas, carcinosarcomas are bigger and more often show ill-defined margins, and can show protrusion into the cervix without extension (Fig. 8).

### Mimickers

Several entities can closely mimic endometrial carcinoma on MRI. Tamoxifen-related endometrial changes are a key pitfall: this drug can induce cystic endometrial hyperplasia and polypoid thickening, often with heterogeneous, sometimes irregular endometrial contours. When typical with a well-defined endometrial–myometrial interface, cancer should be ruled out. Benign endometrial polyps are also frequent mimickers. They typically present as well-circumscribed intracavitary masses arising from the endometrium, sometimes with a fibrovascular core, showing intermediate T2 signal and mild–moderate enhancement (Fig. S13). Small polyps can be indistinguishable from early-stage endometrioid carcinoma on the basis of thickness criteria alone; therefore, a careful multi-parametric analysis combined with a targeted hysteroscopic biopsy is essential to avoid overcalling malignancy.

## Cervical cancer

### Typical presentation

The radiological evaluation of cervical cancer has been standardized by the 2018 FIGO staging. Treatment options are guided by this classification, with local surgery generally limited to early stages (FIGO stage up to IB2 and IIA) [41]. On imaging, cervical cancer typically presents as a relatively T2-hyperintense mass that disrupts the normal low signal intensity of the fibrous cervical stroma. The tumor characteristically exhibits diffusion restriction (manifesting as a high DWI signal and low values on ADC maps), which significantly improves the detection of small lesions. Accurate assessment of parametrial invasion is crucial, as it is generally considered a contraindication to primary surgical treatment. It should be evaluated on T2W images obtained in a plane perpendicular to the long axis of the cervix, looking for disruption of the low-signal-intensity cervical stromal ring and possible direct extension into the parametrium [42] (Fig. S14).

During follow-up, MRI is also fundamental to distinguishing recurrence from fibrotic tissue. Fibrosis tends to present with a low T2 signal and low DWI signal, but occasionally presents with an intermediate T2 signal. In contrast, recurrence typically presents as a lesion with an intermediate T2 signal, restricted DWI signal, and early enhancement on dynamic contrast injection [43].

### Atypical presentations

Gastric-type endocervical adenocarcinoma and its variant, minimal deviation adenocarcinoma (MDA), mostly present as solid infiltrative lesions, but in some cases manifest as multicystic lesions, particularly treacherous on MRI, as they present with a distinctive “honeycomb” or multiloculated cystic appearance, representing the abundant mucin production within the neoplastic glands. Their solid infiltrative pattern tends to be underestimated on MRI because the tumor can appear as a highly subtle lesion with signal characteristics that blend into normal tissue on standard T2W and DWI sequences [44]. Postcontrast fat-saturated T1W sequence is essential, as it allows a better assessment of the extension of the lesion, which enhances poorly compared to normal cervical and myometrial tissue (Fig. S15).

### Mimickers

Endometrial and myometrial tumors are possible pitfalls of cervical lesions, as they can protrude and/or infiltrate the cervical space. Identifying the true epicenter of the mass and possible specific features depending on the tumor can help resolve this pitfall (Fig. S16).

Cervical inflammation represents a significant diagnostic pitfall in the evaluation of cervical lesions, often leading to a false suspicion of advanced malignancy. Cervical actinomycosis, for example, is an uncommon entity that may show limited or no cervical infiltration, yet it exhibits the same features as typical cervical carcinoma: an intermediate T2 signal, high DWI signal with ADC restriction, and even early enhancement on dynamic contrast injection [45, 46] (Fig. S17). Vaginal endometriosis represents another potential pitfall, possibly mimicking cervical cancer in T2W sequences. The presence of an endometriotic signal on the T1W sequence is a reliable feature to suggest endometriosis and not a cervical primary lesion (Fig. S3).

## Adnexal/pelvic masses and ovarian cancer

### Pearls and atypical presentations—O-RADS MR score

The pivotal step in the O-RADS MR risk stratification system is the definitive identification and characterization of solid tissue (e.g., papillary projections, mural nodules, or solid components). Dynamic Contrast-Enhanced (DCE) imaging represents the cornerstone of this evaluation, outperforming subjective visual assessment [3, 47].

- Time-intensity curve (TIC) Type 1 demonstrates a progressive, gradual increase without a plateau. This pattern carries a high negative predictive value for invasive malignancy and categorizes a lesion as O-RADS MR 3.
- TIC Type 2 (O-RADS MR 4) demonstrates an initial progressive increase followed by a plateau.
- TIC Type 3 (O-RADS MR 5), on the other hand, displays a sharp initial uptake steeper than the external myometrium, followed by a washout phase, which is highly suggestive of malignancy.

**Fig. 9:**
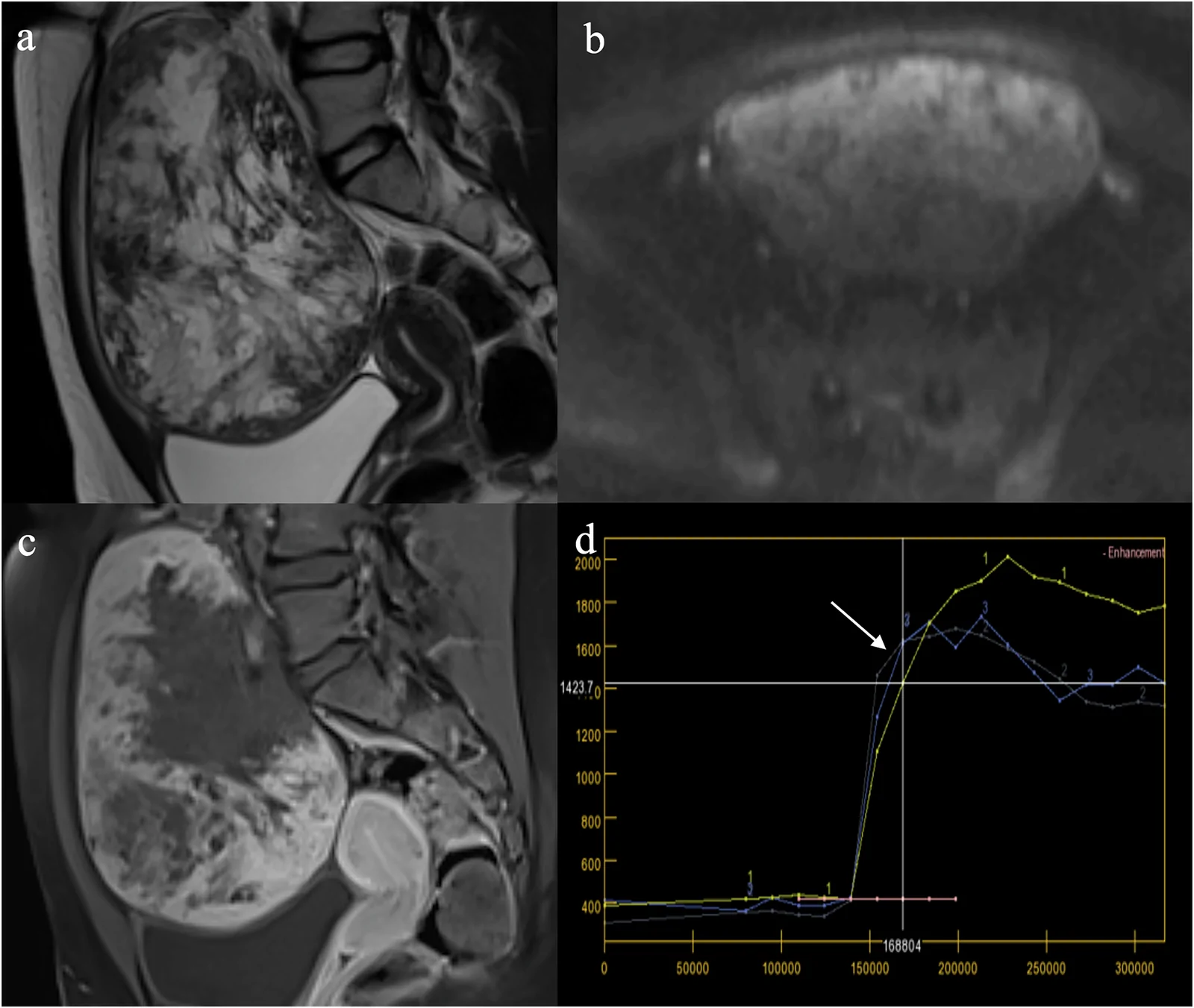
Example of benign O-RADS MR 5 tumor. **a** Sagittal T2-weighted sequence; **b** axial DWI sequence; **c** sagittal postcontrast fat-saturated T1-weighted sequence; **d** TIC from DCE MR sequence. A young patient (20 years old) is referred for left ovarian lesion characterization. The images show a large left ovarian tumor with heterogeneous signal intensity, including areas of high T2W signal, and high diffusion signal. Postcontrast imaging shows a large central necrotic area. TIC from DCE MR sequence shows a high-risk time-intensity curve (blue curve with white arrow) with a particular washout. Lesion is classified as O-RADS MR 5. Given the young age of the patient and the presence of a typical washout pattern on TIC, a sclerosing stromal tumor was suspected. Conservative surgery was performed, and pathology confirmed the diagnosis

TIC analysis is also useful for advanced tissue characterization; for example, in the presence of a purely solid tumor with low T2 signal, a Type 1 TIC suggests a fibroma, whereas a Type 2 TIC points toward a Brenner tumor (Fig. S18). Radiologists must remain vigilant regarding benign hypervascular exceptions. Highly cellular benign tumors, specifically sclerosing stromal tumors and cellular fibrothecomas, frequently exhibit early, avid contrast medium uptake and a washout pattern (Fig. 9). This mimics high-risk malignant behavior despite their histologically benign nature [48].

**Fig. 10:**
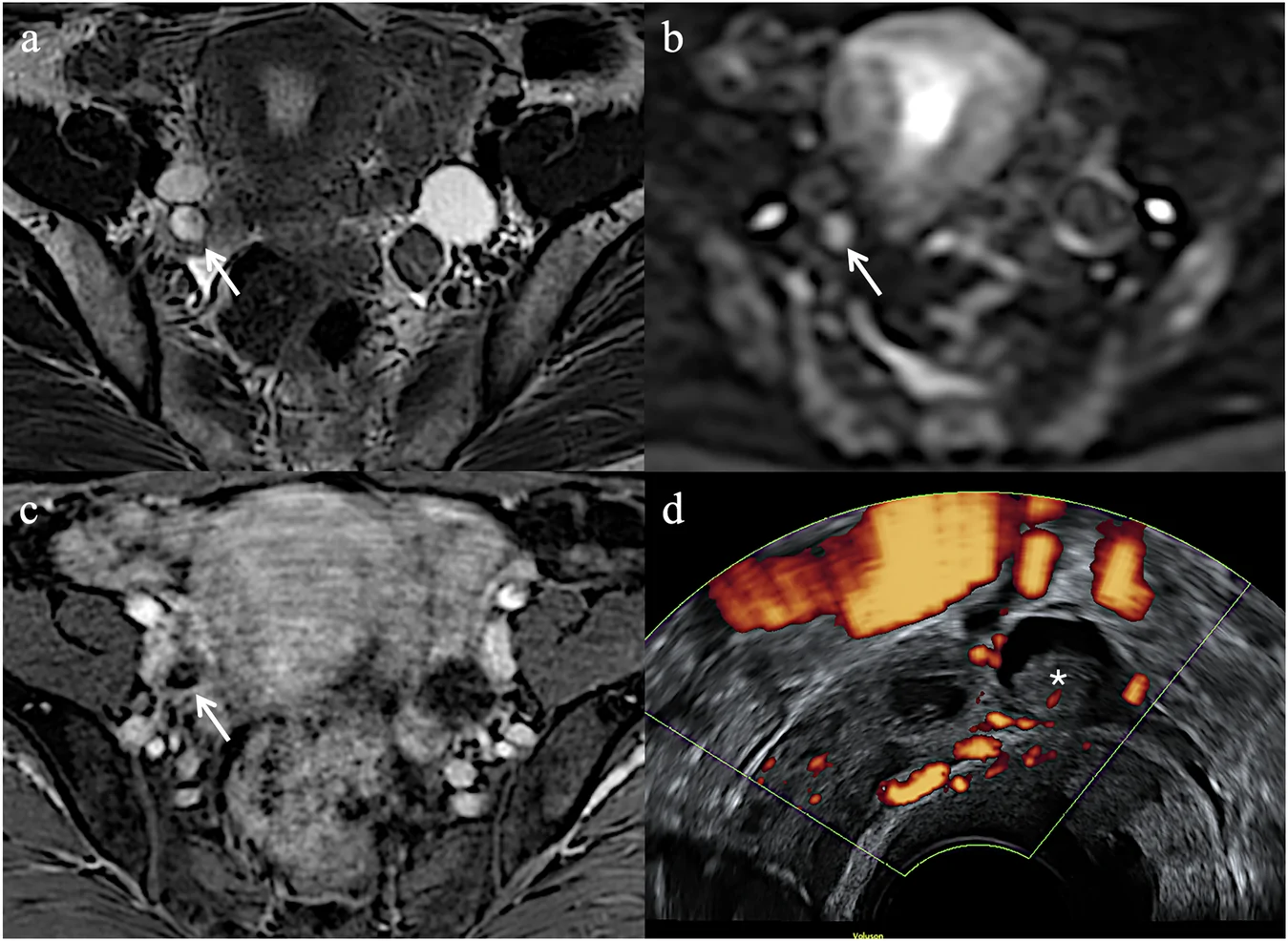
Impact of second-look ultrasonography. **a** Axial T2-weighted sequence; **b** axial DWI; **c** axial postcontrast fat-saturated T1-weighted sequence; **d** second-look ultrasonography. Case of a 25-year-old patient referred for cystic adnexal lesion characterization. MRI shows a right cystic locule with intermediate T2W signal and high diffusion signal (white arrow). The postcontrast sequence shows equivocal enhancement within the locule. Second-look ultrasonography confirmed a papillary projection (*). The lesion was classified as O-RADS MRI score 4, and a borderline serous tumor was suspected. Surgical management confirmed the diagnosis. This case highlights the potential value of second-look ultrasonography in detecting small solid tissue

Specific patterns within the O-RADS 2 and 3 categories require additional nuances:

- High T1W signal intensity within a cyst is not pathognomonic for a simple endometrioma. If any internal enhancement is detected via subtraction sequences, the lesion can no longer be classified as O-RADS MR 2. For this purpose, subtraction is vital to differentiate simple blood products from decidualized cysts during pregnancy or Endometriosis-Associated Ovarian Cancer (EAOC) [4].
- In the context of mature cystic teratomas (O-RADS MR 2), the Rokitansky protuberance should not be mistakenly overcalled as a malignant solid nodule. It may display intense enhancement on DCE sequences due to functional thyroid tissue components (struma ovarii), and should not be classified based on the DCE sequence alone (Fig. S19) [49]. Fat-poor teratomas lack a clear macroscopic signal drop on standard fat-suppressed sequences. To prevent misclassifying them as complex non-fatty masses, radiologists should search for micro-calcifications, developing teeth, or subtle fat-fluid levels on unenhanced T1W Dixon phases. Conversely, immature teratomas should be suspected when a solid tissue component larger than 5 cm with irregular margins, including punctate foci of adipose tissue, is present within a multicystic mass [50].
- Highly viscous proteinaceous fluid or unorganized blood clots can easily simulate solid tissue by showing intermediate T2 signal, moderate T1 hyperintensity, and restricted diffusion. Image subtraction is mandatory to rule out enhancement, allowing a unilocular proteinaceous cyst to be safely categorized as O-RADS MR 3 [48]. If a true solid component demonstrates low signal intensity on both T2W and high b-value DWI sequences, it reflects dense fibrous tissue. Such lesions remain O-RADS MR 2, even when discovered within a multilocular mass. When thin, irregular septations or tiny intracystic elements yield equivocal MR findings, a second-look transvaginal ultrasound (TVUS) can leverage superior spatial resolution to confirm or rule out true solid papillary projections (Fig. 10).

Quantitative analysis of the Apparent Diffusion Coefficient (ADC) is a powerful ancillary tool, but it should not be used to score O-RADS, as ADC values overlap significantly between benign lesions and invasive cancers [51]. Instead, ADC mapping should be reserved to stratify lesions initially categorized as O-RADS MR 4 mixed masses [52]. While purely solid masses should not be sub-stratified this way, an ADC threshold of approximately 1.08 × 10⁻³ mm²/s offers excellent specificity: values below this suggest invasive malignancy, whereas higher values point toward borderline or benign pathology.

True O-RADS MR 5 lesions are dominated by solid, avidly enhancing masses, peritoneal carcinomatosis, and pelvic metastases (often arising from gastrointestinal primaries). However, acute inflammatory processes can easily simulate this category. For instance, tubo-ovarian abscesses (TOAs) often present as complex masses with thick, intensely enhancing walls, purulent fluid restriction, and prominent surrounding pelvic fat stranding. Strict correlation with clinical symptoms (fever, pelvic pain) and inflammatory biomarkers is mandatory to avoid mistaking a TOA for advanced ovarian cancer.

Finally, when a patient presents with acute pelvic pain and imaging reveals signs of adnexal torsion, the O-RADS MR risk stratification model must not be applied (Fig. S20). Massive hemorrhagic infarction and compromised tissue perfusion completely distort normal enhancement patterns, rendering the O-RADS score unreliable. In these scenarios, urgent surgical management must be recommended.

### Pearls and atypical presentations—pathological hypothesis

While O-RADS classification is a crucial first step, the prediction of pathological subtypes is essential, particularly during MDT sessions if surgical management is being considered [53].

**Fig. 11:**
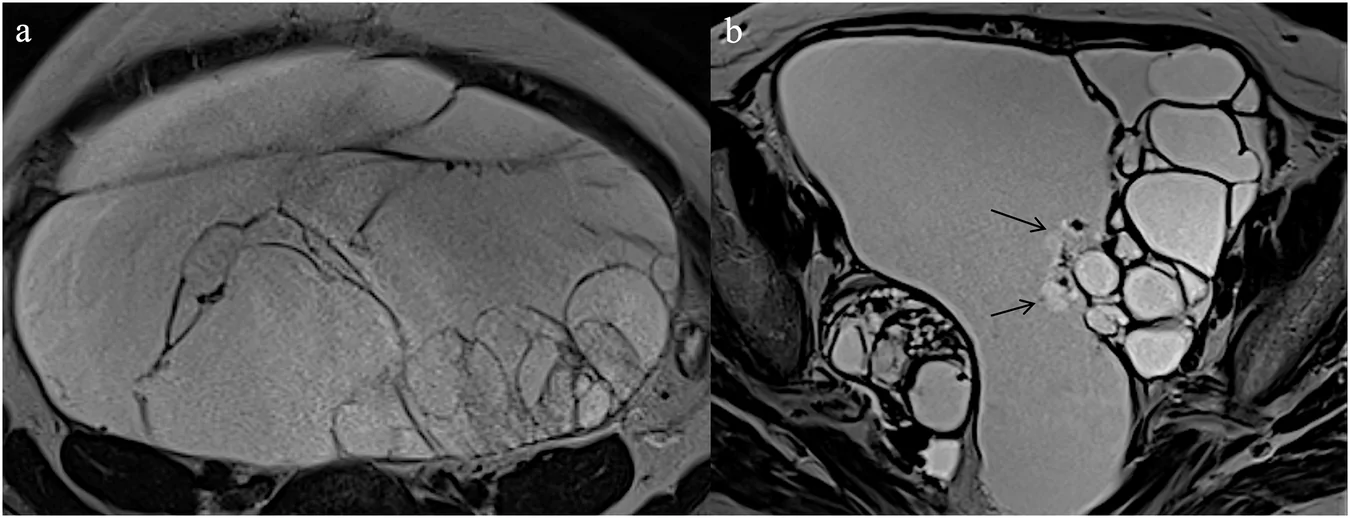
Mucinous borderline tumor and cystadenofibroma. **a**, **b** Axial T2-weighted sequences. **a** A typical mucinous borderline tumor and **b** a typical cystadenofibroma. Both appear as large multicystic tumors. Cystadenofibroma specifically shows low-T2 fibrous tissue, thickened regular fibrous septa, and papillary projections (black arrows), with bilateral involvement

Certain entities present typical contrast-enhancement patterns that assist in classification. Cystadenofibromas demonstrate low-T2 fibrous tissue, thickened regular fibrous septa, often bilateral involvement, and may sometimes exhibit papillary projections (Fig. 11). Cystadenofibromas may be classified O-RADS 2, 3, or 4. The rating according to the O-RADS system gives, in addition to the pathological hypothesis, a degree of certainty [54]. Table 3 presents the different pathological hypotheses classified according to their O-RADS score, morphological features, and pathological subtypes to help the readers in making differential diagnosis.

**Table 3:**
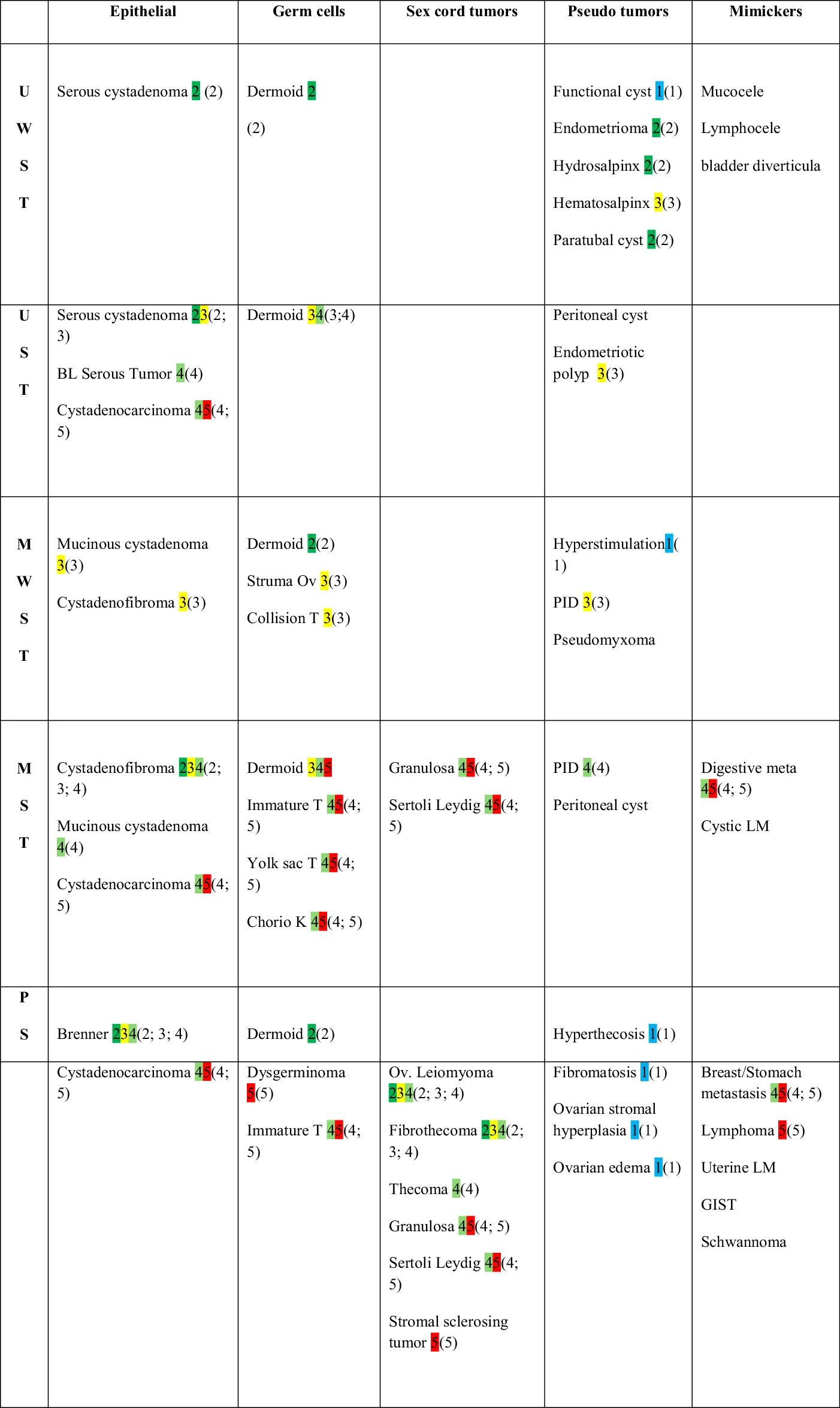
O-RADS and pathological correlation to MR morphological and functional features

Color code corresponds to O-RADS MR Classification 5 , 4 , 3 , 2 , 1

*UWST* unilocular without solid tissue, *UST* unilocular with solid tissue, *MWST* multilocular without solid tissue, *MST* multilocular with solid tissue, *PS* purely solid

### Adnexal masses and ovarian cancer mimickers

Mimickers of adnexal pathology must be carefully considered, as they may lead to erroneous O-RADS MR attribution. More than 10% of women referred for MRI to characterize a TVUS adnexal mass are ultimately found to have a non-adnexal lesion [55]. Determining the origin is a frequent cause of misdiagnosis in adnexal masses, accounting for up to 20% of all diagnostic errors [56]. Determining whether a pelvic lesion is adnexal relies primarily on identifying a structural connection with the ovary. The most reliable criterion is direct continuity with ovarian parenchyma, where the lesion appears to arise from, deform, or partially replace normal ovarian tissue. The presence of residual follicles along the periphery of the mass, creating the classic “claw” or “crescent” sign of compressed functional cortex, strongly supports an ovarian origin [57]. Another robust feature is attachment to the utero-ovarian ligament or the suspensory (infundibulopelvic) ligament, which confirms adnexal vascular and ligamentous continuity.

When these specific features cannot be demonstrated, several secondary—but less specific—elements may still suggest adnexal origin. These include the presence of papillary projections or a predominantly cystic internal architecture, which are commonly encountered in ovarian neoplasms, while a solid tissue enhancement similar to that of the myometrium favors uterine origin, especially for solid masses [58]. Although these findings are not definitive by themselves, their association with typical adnexal topography may help classify the lesion as ovarian when direct connection is difficult to demonstrate.

Score 1 mimickers include unilocular appendiceal mucoceles, which may closely resemble simple cystic adnexal lesions, and multilocular peritoneal inclusion cysts typically encountered in patients with a history of surgery, infection, or endometriosis (Fig. S21). Solid mimickers encompass ovarian stromal hyperplasia or ovarian fibromatosis after ovarian biopsies or surgery (Fig. S22) (frequently bilateral), ovarian abscess, and a variety of extra-ovarian masses such as pedunculated subserosal leiomyomas recognizable by the presence of the bridging vessel sign and claw or beak configurations (Fig. S23).

**Fig. 12:**
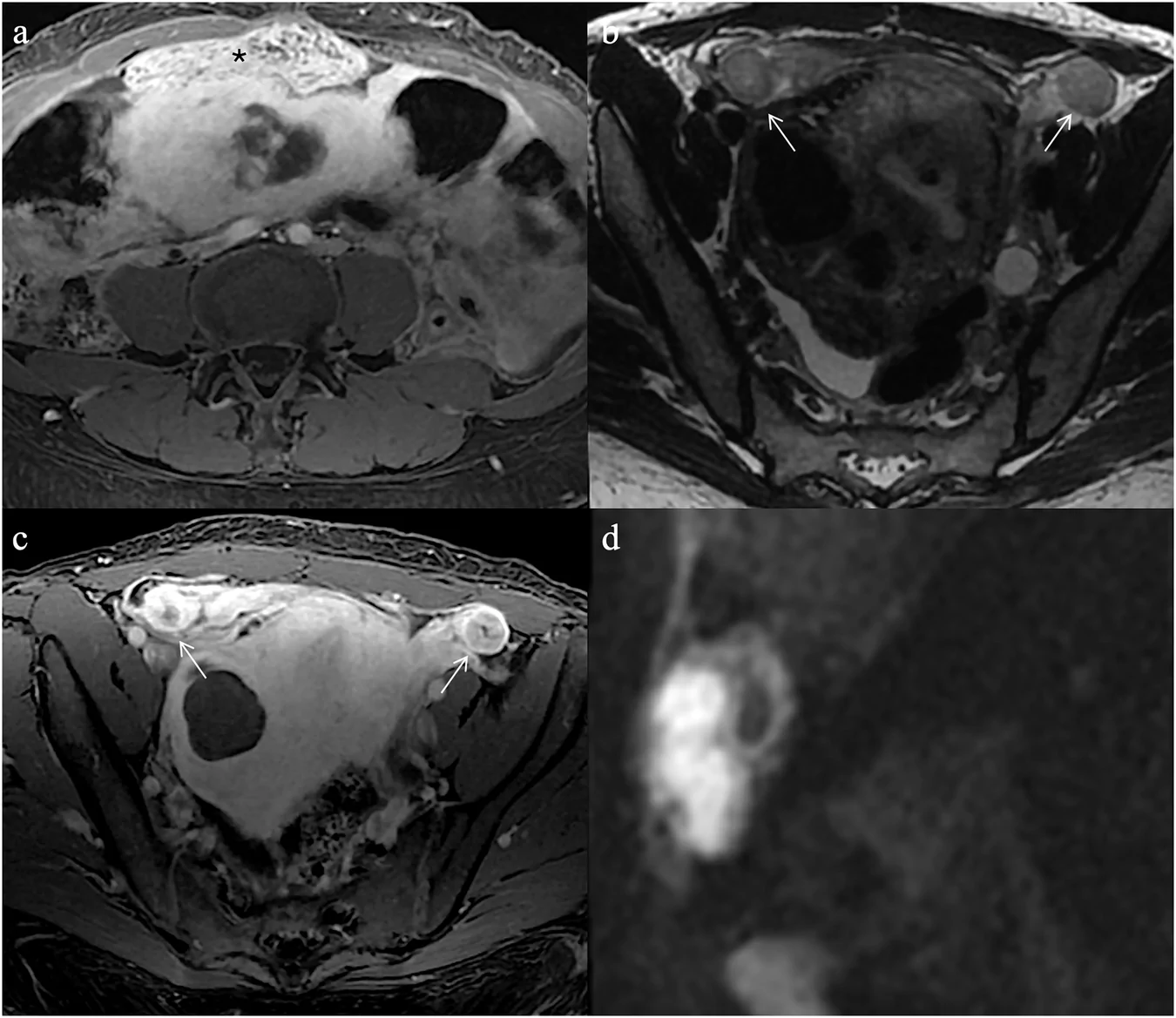
Peritoneal tuberculosis. **a** Axial postcontrast fat-saturated T1-weighted sequence; **b** axial T2-weighted sequence; **c** axial postcontrast fat-saturated T1-weighted sequence; **d** sagittal DWI sequence centered on the right fallopian tube. A 40-year-old patient was admitted for exploration of peritoneal implants in the context of subacute fatigue with night sweats. The images show omental thickening and enhancement (*), associated with tubal thickening with marked enhancement (white arrows). On diffusion, the right fallopian tube shows marked diffusion signal intensity, suggesting purulent content. Peritoneal tuberculosis was subsequently confirmed

Conversely, score 5 mimickers correspond to entities that may falsely suggest advanced malignancy. In such cases, combined interpretation of T2W images and wide-field diffusion imaging is essential. False positives include peritoneal tuberculosis (Fig. 12), disseminated leiomyomatosis (Fig. S24), peritoneal mesothelioma, and splenosis, all of which may mimic nodular peritoneal implants. Accurate differentiation therefore relies on careful correlation with imaging characteristics, anatomic origin, and clinical context. Conversely, calcified peritoneal carcinomatosis may appear falsely negative on diffusion, while remaining highly suggestive on CT, particularly in low-grade serous carcinoma.

## Conclusion

In conclusion, standardized protocols and structured interpretation significantly improve diagnostic accuracy. Gynecological pelvic MRI provides radiologists with an advanced tool for differential diagnosis, thanks to its exceptional ability to characterize both cystic and solid tissues in detail. Diagnostic pitfalls and mimickers are numerous and must be thoroughly understood to avoid misdiagnosis.

## Supplementary information

Electronic Supplementary Material

## Data Availability

Data and materials are available upon request.
