# Supplementary material for: Gynecologic imaging errors and pitfalls: how to avoid misdiagnosis on pelvic MRI
Source: Insights Imaging. 2026 Sep 17;17:229. doi: 10.1186/s13244-026-02378-2 (PMC13586104; doi:10.1186/s13244-026-02378-2)

Gynecologic imaging errors and pitfalls: How to avoid misdiagnosis on pelvic MRI

Electronic Supplementary Material

Figure S1: Endometrioma rupture with clot.

a) Sagittal T2-weighted sequence; b) and d) axial T2-weighted sequence; c) and e) axial T1 water-only images from a T1-weighted Dixon sequence; These images show a ruptured endometrioma with a dependent clot in the context of deep pelvic endometriosis (arrow). The patient had a history of multiple punctures in the setting of infertility.

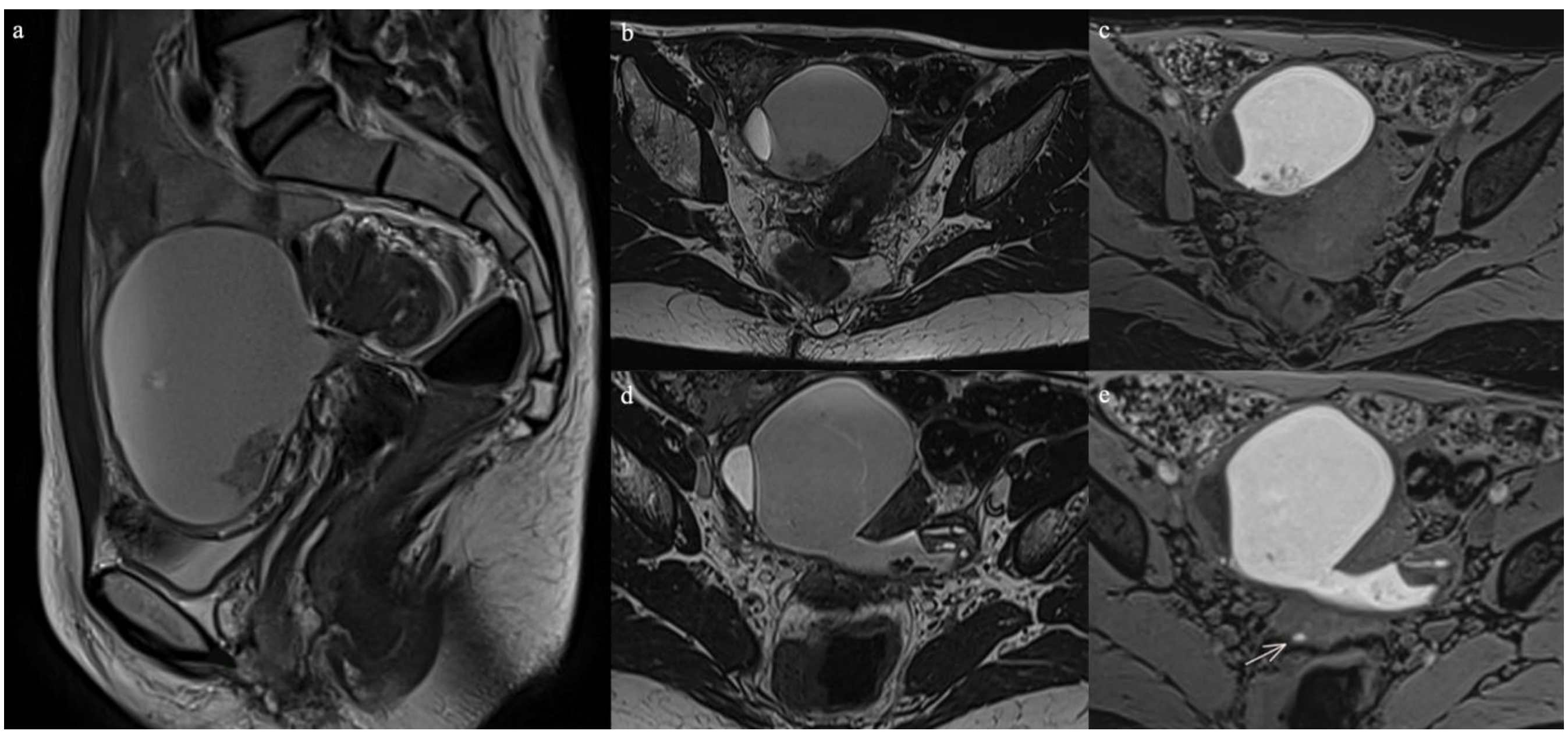

Figure S2: Endometrioma decidualization

a) and b) ultrasonography with color Doppler; c) axial T2-weighted sequence; d) axial T1 water-only image from a T1-weighted Dixon sequence. A pregnant patient referred for MRI because of a left ovarian lesion detected on ultrasonography performed on the same day. Initial ultrasonography shows a large predominantly cystic left ovarian lesion with mural nodules demonstrating vascularity on color Doppler. MRI shows a left ovarian cyst with endometriotic content (*) with mural nodules of relatively high T2 signal. Anterior to the lesion, the uterus is visible with the fetal head partially seen. A decidualized endometrioma was suspected, and careful follow-up was performed. The lesion progressively decreased in size, confirming the diagnosis.

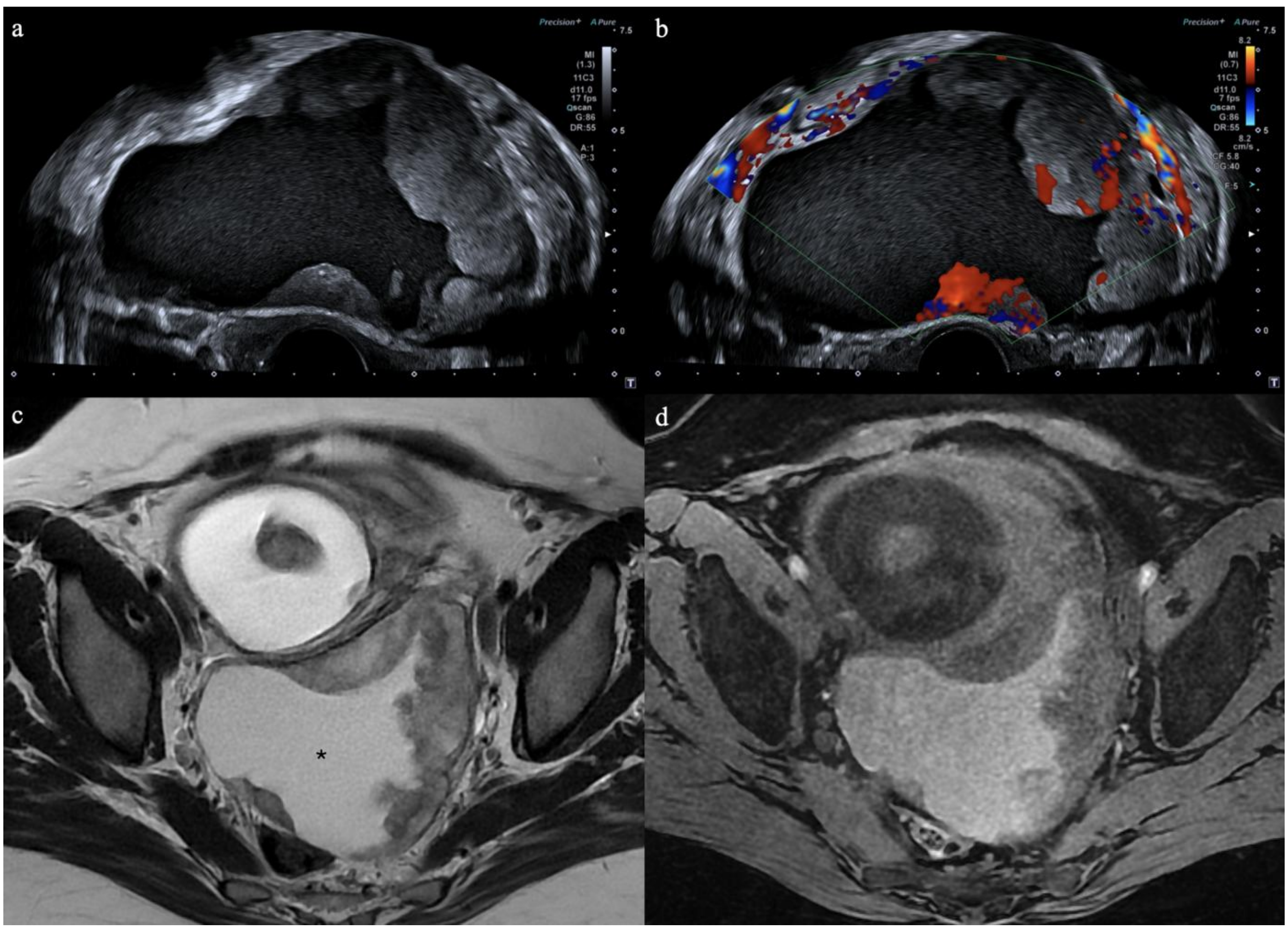

Figure S3: Naboth cyst and vaginal endometriotic locations

a) sagittal T2 weighted sequence; b) axial T2 weighted sequence; c) sagittal T1 water-only image from a T1-weighted Dixon sequence. d) axial T1 water-only image from a T1-weighted Dixon sequence; The images show Naboth cysts which are central and high T2W signal, low T1W signal (thin arrow) and vaginal endometriotic infiltration with a low T2W thickening and high T1W signal.

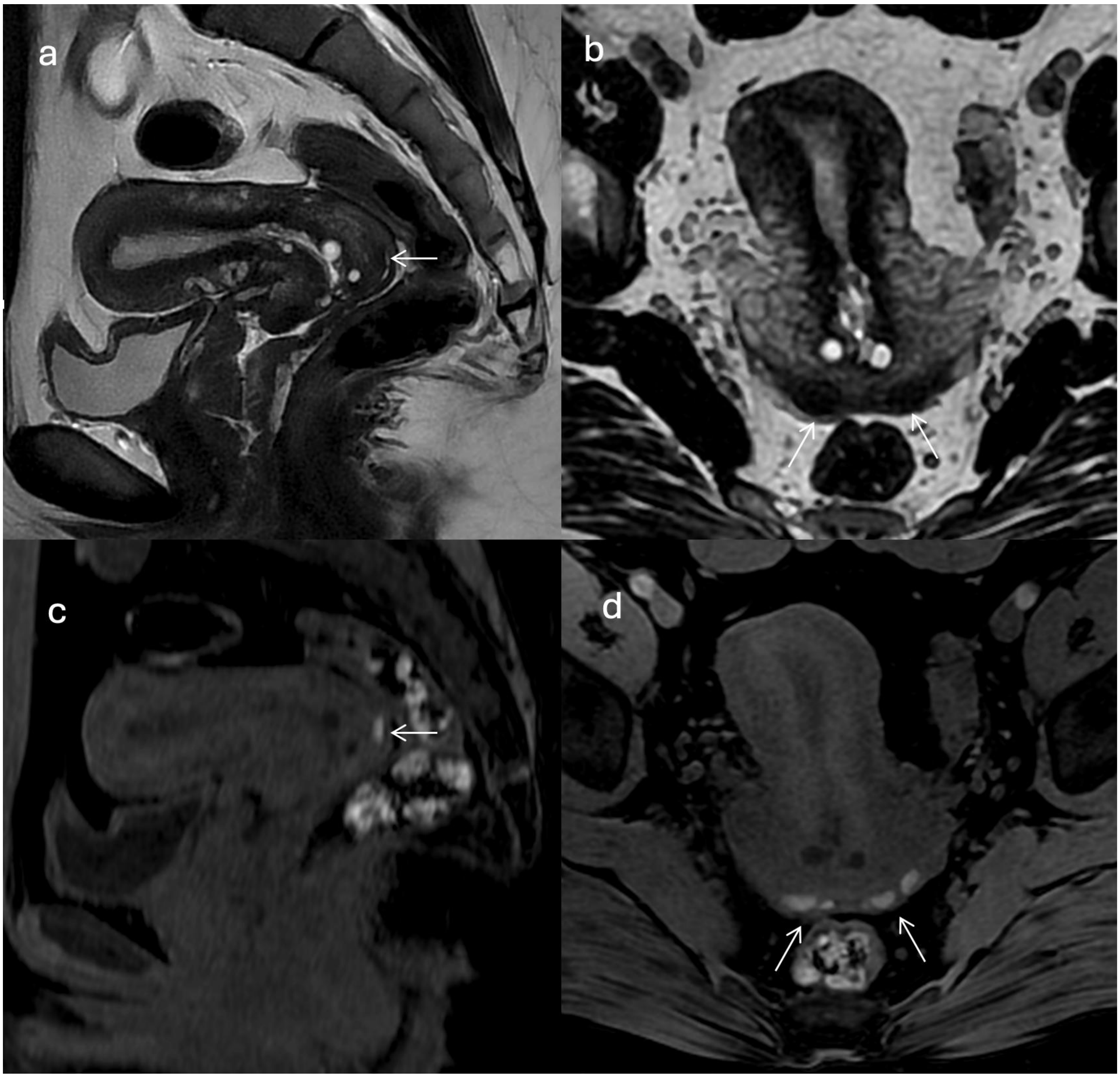

Figure S4: Feces artifact

a) axial T1 water-only image from a T1-weighted Dixon sequence; b) Sagittal T2-weighted sequence; c) coronal T2-weighted sequence. Normal appendix filled with feces in a 30-year-old woman with dysmenorrhea and medical history of endometriosis. The images show that the T1W "spot" (white arrow) is located in a tubular structure (c) corresponding to the appendix running behind the right ovary (black arrow), consistent with endoluminal feces concretion.

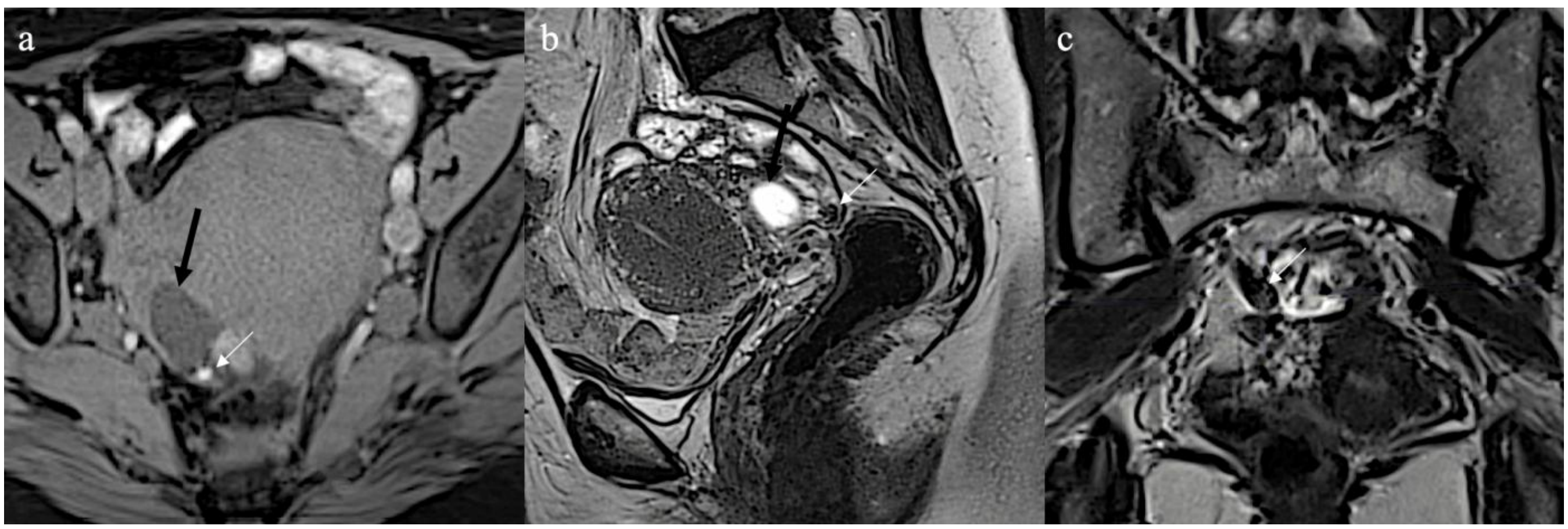

Figure S5: Vascular artifact

Flow-related enhancement artifact, also known as “the entry slice phenomenon”, in a 27-year-old woman with chronic pelvic pain and dysmenorrhea.

a) axial T1 water-only image from a T1-weighted Dixon sequence; b) Axial T2-weighted sequence. A 27-year-old patient was admitted for chronic pelvic pain and dysmenorrhea. The MR images show a bright T1W spot (black arrow) close to the ovarian fossa. Axial T2-weighted MR image shows an enlarged vein (black arrow) in place of the T1W bright spot, behind the ovary (white arrow) and with other varicose veins (broken white arrow).

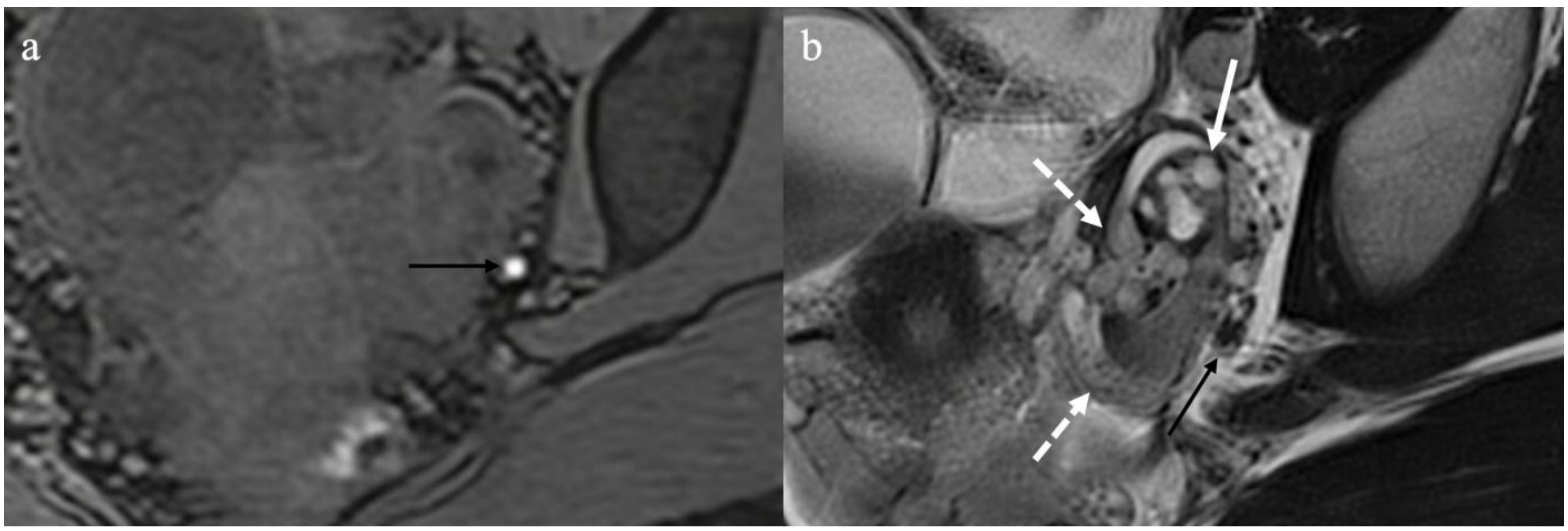

Figure S6: Actinomycosis in a 32-year-old woman with chronic pelvic pain, rectal disorder and dyspareunia.

a) Axial T2-weighted sequence; b) axial T1 water-only image from a T1-weighted Dixon sequence; c) post-contrast fat-saturated T1-weighted sequences. The images show left peritoneal and subperitoneal infiltration with intermediate T2W and intermediate T1W signal intensity, centered on the right utero-sacral ligament (star) and infiltrating the left adnexa (dotted arrow) and thickening of the mid-anterior rectum (long arrow). Note the thickening and infiltration of the left round ligament up to the inguinal canal (short arrows). After injection, image shows avid enhancement of the surrounding inflammatory infiltration with abscesses (*). A recent medical history of intrauterine device removal was found.

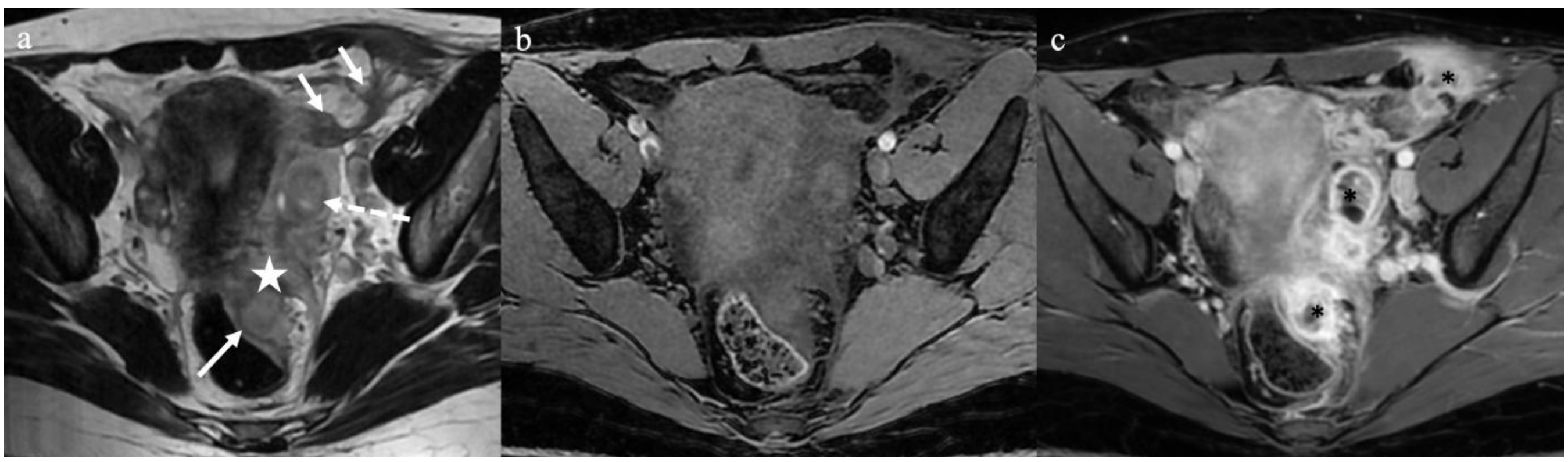

Figure S7: Unilateral right-sided vesicoureteral reflux surgical bulking agent in a 20-year-old woman.

a) sagittal T2-weighted sequence; b) axial T2-weighted sequence; c) axial T1 water-only image from a T1-weighted Dixon sequence. The MR images show an ovoid, geometrically shaped, T2W hypointense unilateral right-sided vesicoureteral reflux implant (black arrows) at the ureterovesical junction with corresponding T1W isosignal intensity (white arrow) without any bright spots.

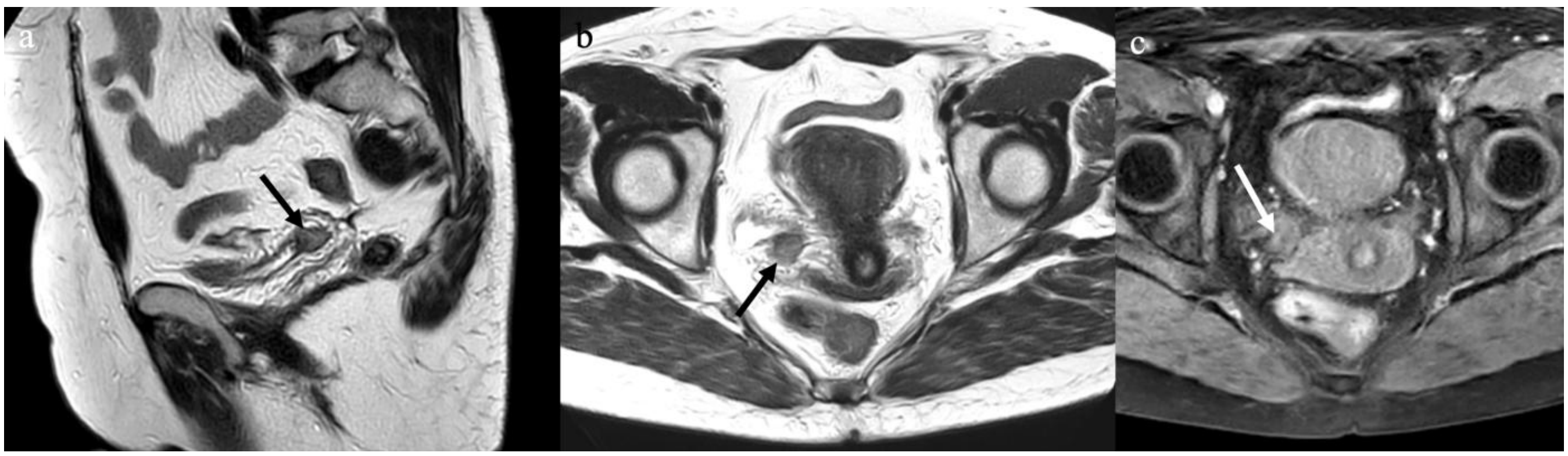

Figure S8: Diffuse and focal adenomyosis:

a) Sagittal T2-weighted image; b) sagittal T1 water-only image from a T1-weighted Dixon sequence. The images show typical diffuse adenomyosis, characterized by diffuse ill-defined thickening of the junctional zone associated with punctate T2- and T1-hyperintense foci and small cysts.

c) Sagittal T2-weighted image; d) sagittal T1 water-only image from a T1-weighted Dixon sequence. The images show focal adenomyosis, characterized by the same features as diffuse adenomyosis, but confined to a focal myometrial area, here in the posterior myometrium.

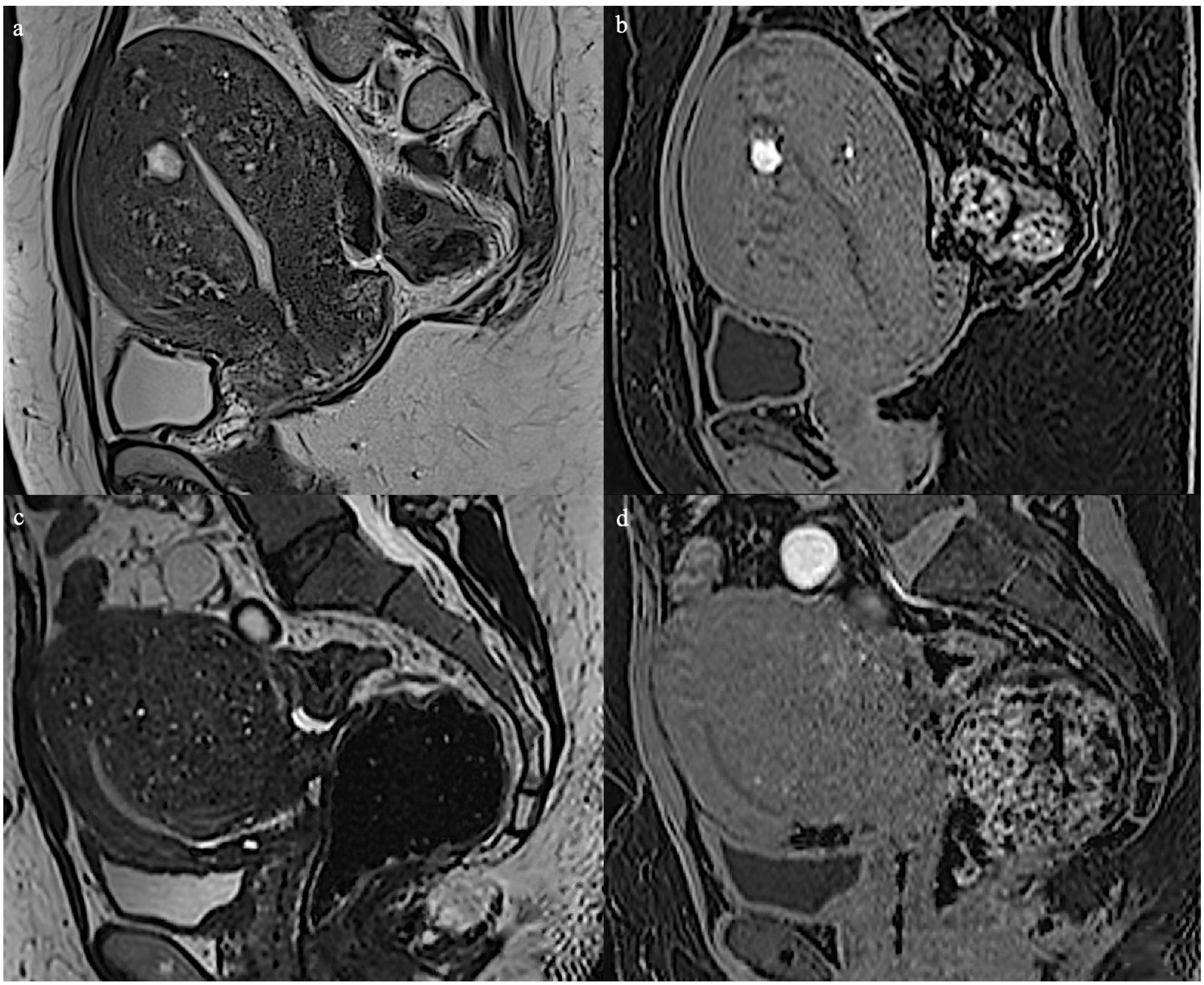

Figure S9: FH deficient leiomyoma in a premenopausal woman.

a) axial T2-weighted sequence; b) sagittal T2-weighted sequence; c) axial DWI sequence; d) axial post-contrast fat-saturated T1-weighted sequence. The images show a well circumscribed relatively homogeneous myometrial lesion with intermediate T2W signal with some microcysts (white arrows), high DWI signal and homogeneous intense enhancement. ADC map (not shown) demonstrated values measured as low as 0.9×10^{-3} mm²/s. Lesion was classified as score 3 according to Zlotykamien-Taieb et al (7).

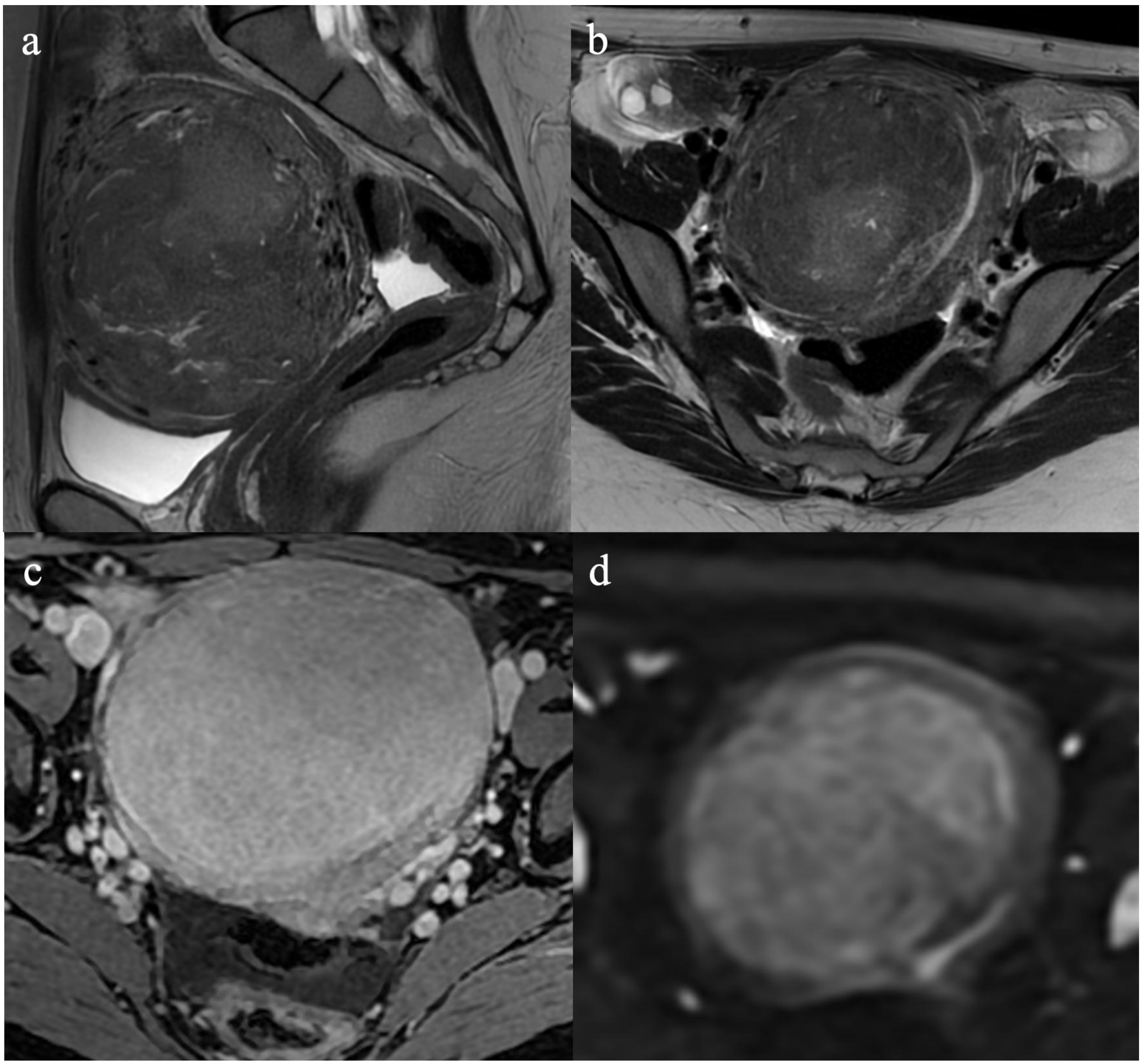

Figure S10: Myometrial tumor mimickers:

a) coronal T2-weighted sequence. The image show a left ACUM (white arrow) which is typically located in uterine horn and always associated with chronic pelvic pain.

b) axial T2-weighted sequence. A second patient with image demonstrating a left interstitial pregnancy (*).

c) sagittal T2-weighted sequence; d) sagittal water-only image from a T1-weighted Dixon sequence. A third patient with images demonstrating a posterior pedunculated adenomyoma with typical endometriotic cysts. Pathology confirmed the diagnosis.

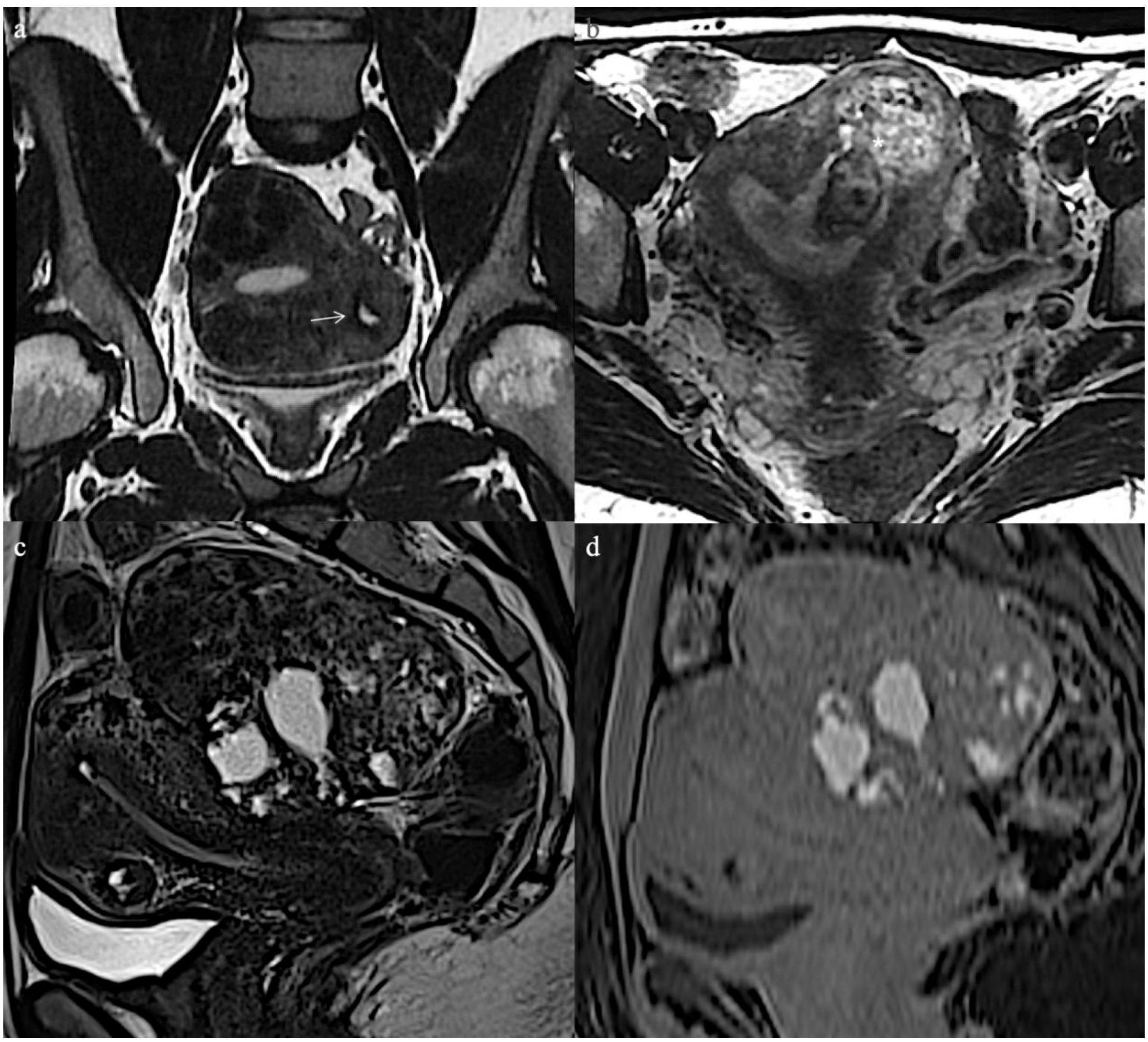

Figure S11: STUMP.

a) axial T2-weighted sequence; b) axial water-only image from a T1-weighted Dixon sequence; c) axial post-contrast fat-saturated T1-weighted; d) DWI sequence. The mages show a heterogenous ill-defined myometrial lesion with intermediate T2W signal, spontaneous T1W signal corresponding to hemorrhage. Post-contrast imaging shows large necrotic areas and infiltrative peripheral parts with relatively high DWI signal. ADC map (not shown) demonstrated values as low as 0.8×10^{-3} mm²/s in the enhancing component. As the patient was premenopausal, the lesion was classified as score 4 (7).

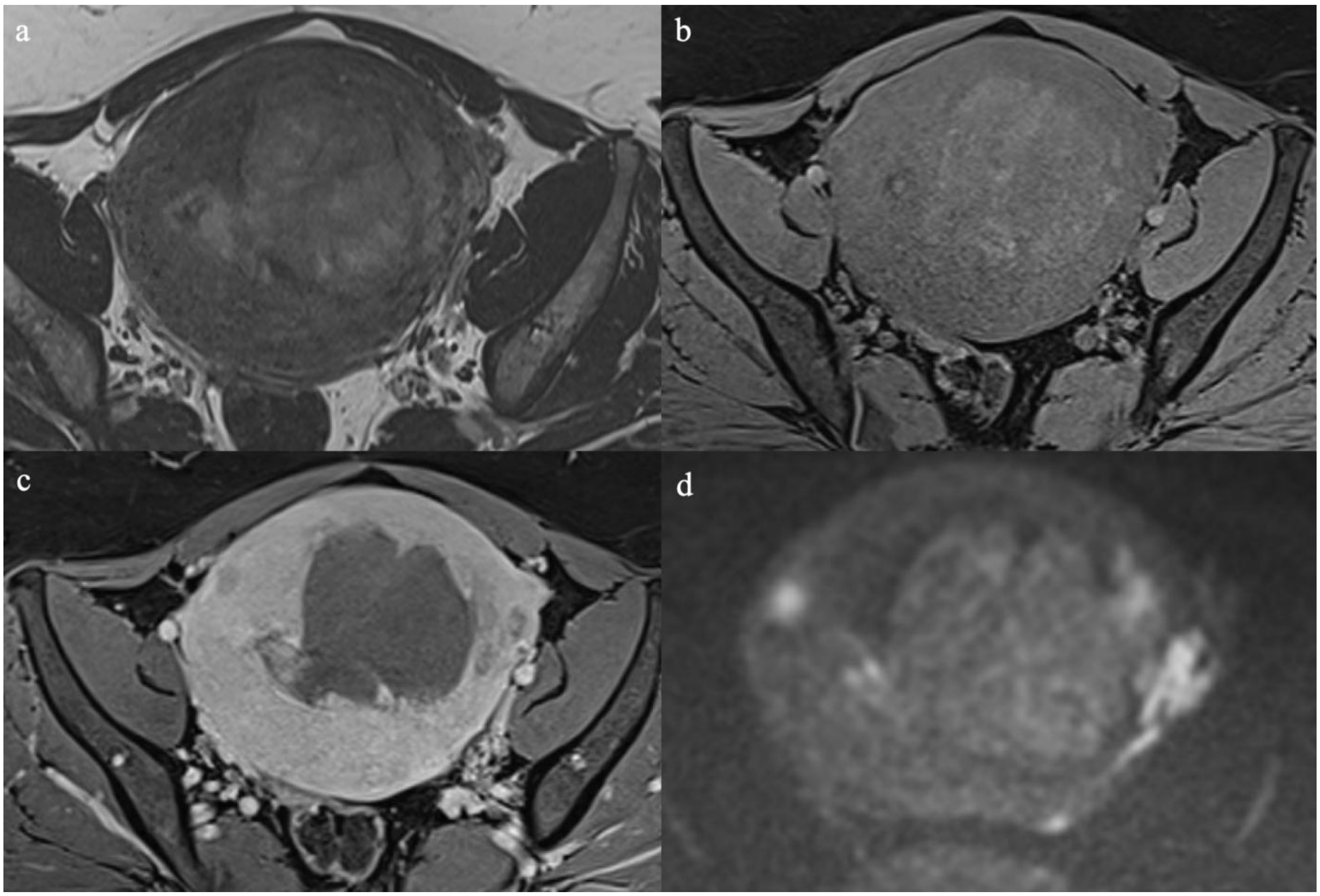

Figure S12: Endometrial tumors; atypical presentations:

a) sagittal T2-weighted sequence; b) axial T2-weighted sequence. The images show a typical FIGO stage IA3 low-grade endometrial carcinoma with unilateral ovarian involvement.

c) sagittal T2-weighted sequence; d) axial T2-weighted sequence. Another patient with a low-grade endometrial carcinoma with the typical “horseshoe” pattern of metastasis around the urethra, sparing the fibrous plane between the urethra and the vagina.

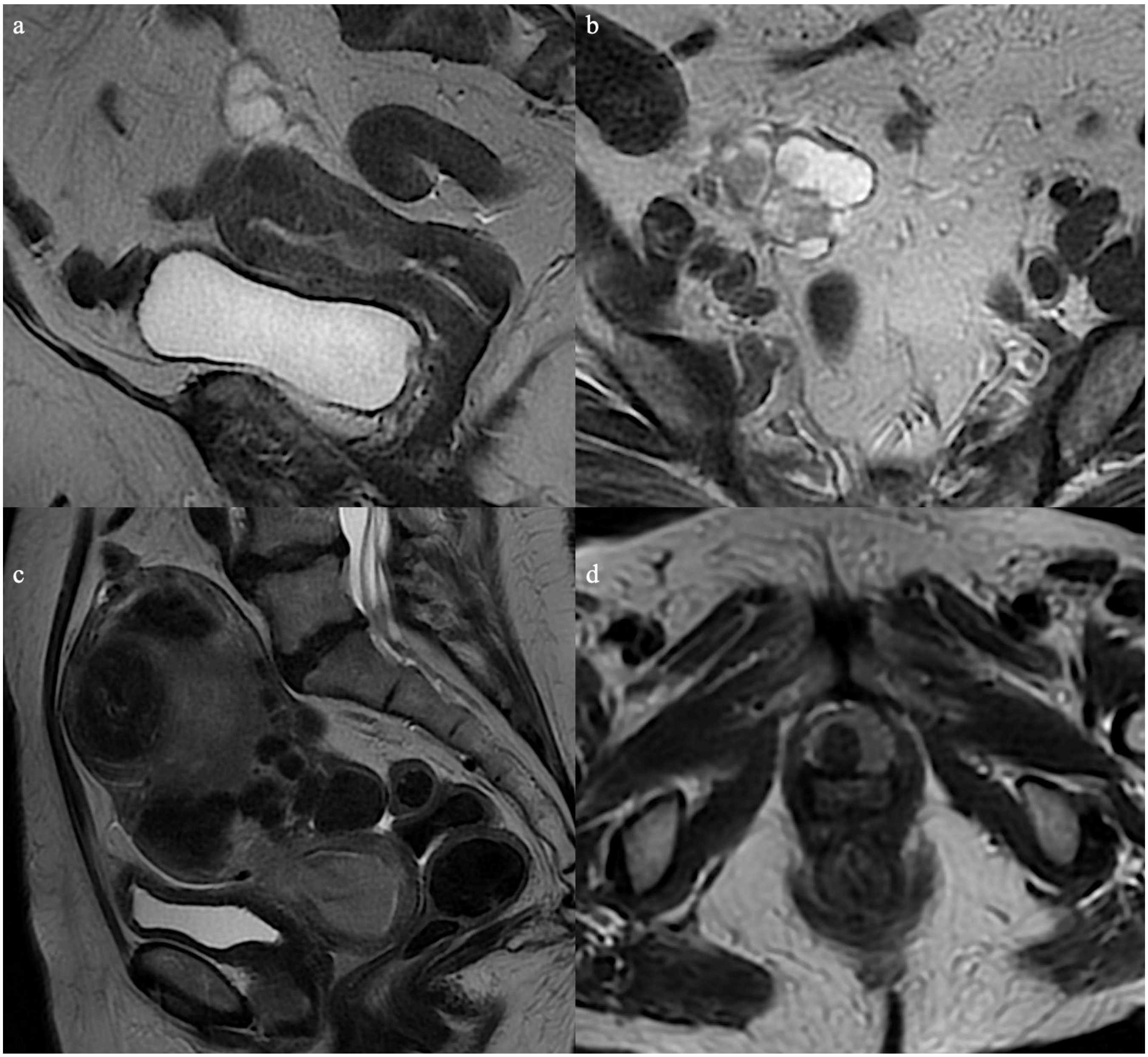

Figure S13: Endometrial tumor mimicker: endometrial polyp

a) sagittal T2-weighted sequence; b) ADC MAP. The images is show an endometrial well defined lesion confined to the endometrium in intermediate T2 signal, visible fibrovascular core, and typically high ADC value (here measured at 2×10^{-3} mm²/s).

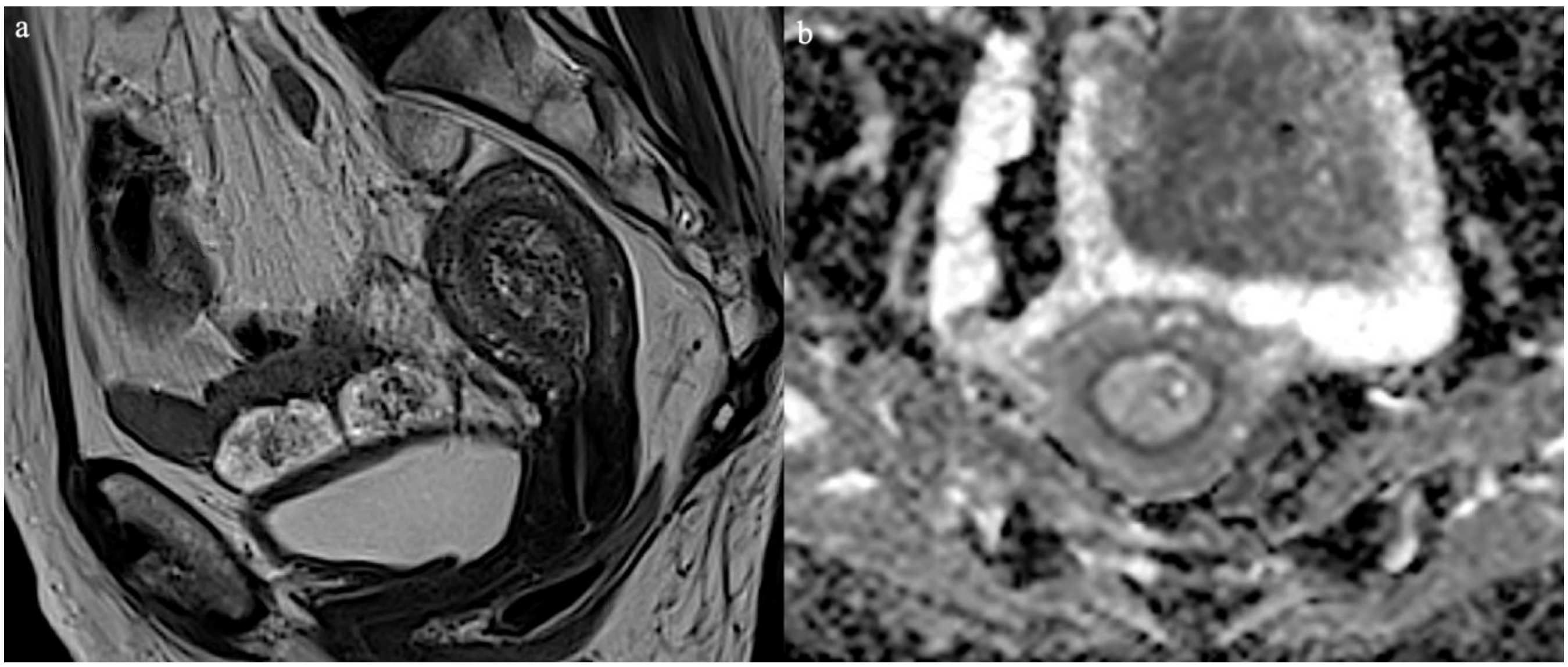

Figure S14: Typical cervical adenocarcinoma

a) sagittal T2-weighted sequence; b) axial T2-weighted sequence; c) sagittal DWI sequence; d) axial post-contrast fat-saturated T1-weighted sequence. A 40-year-old patient was referred for locoregional staging of cervical adenocarcinoma. MRI shows a large infiltrative cervical lesion (*) with intermediate T2 signal intensity, high signal on diffusion-weighted imaging, and post-contrast enhancement. On axial T2-weighted imaging, there is focal disruption of the left cervical stromal ring (white arrow) and parametrial extension. No lymph node involvement was identified, consistent with FIGO stage IIB.

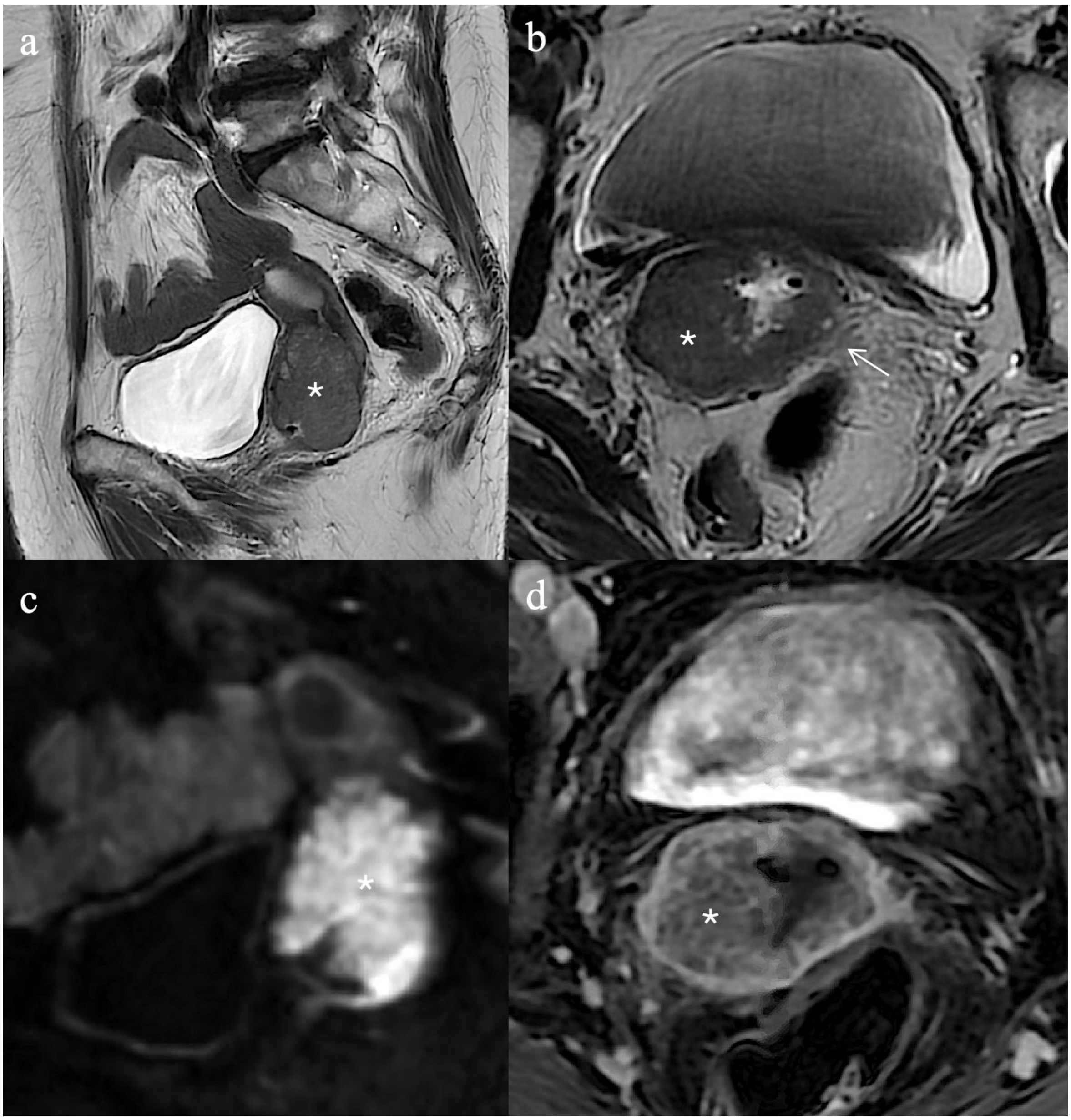

Figure S15: Cervical mucinous carcinoma

a) sagittal T2-weighted sequence; b) sagittal DWI sequence; c) sagittal post-contrast fat-saturated T1-weighted sequence. MRI shows an ill-defined cervical lesion, poorly visible on T2-weighted imaging, not visible on diffusion-weighted imaging, and demonstrating only mild enhancement. Pathological examination confirmed cervical mucinous carcinoma.

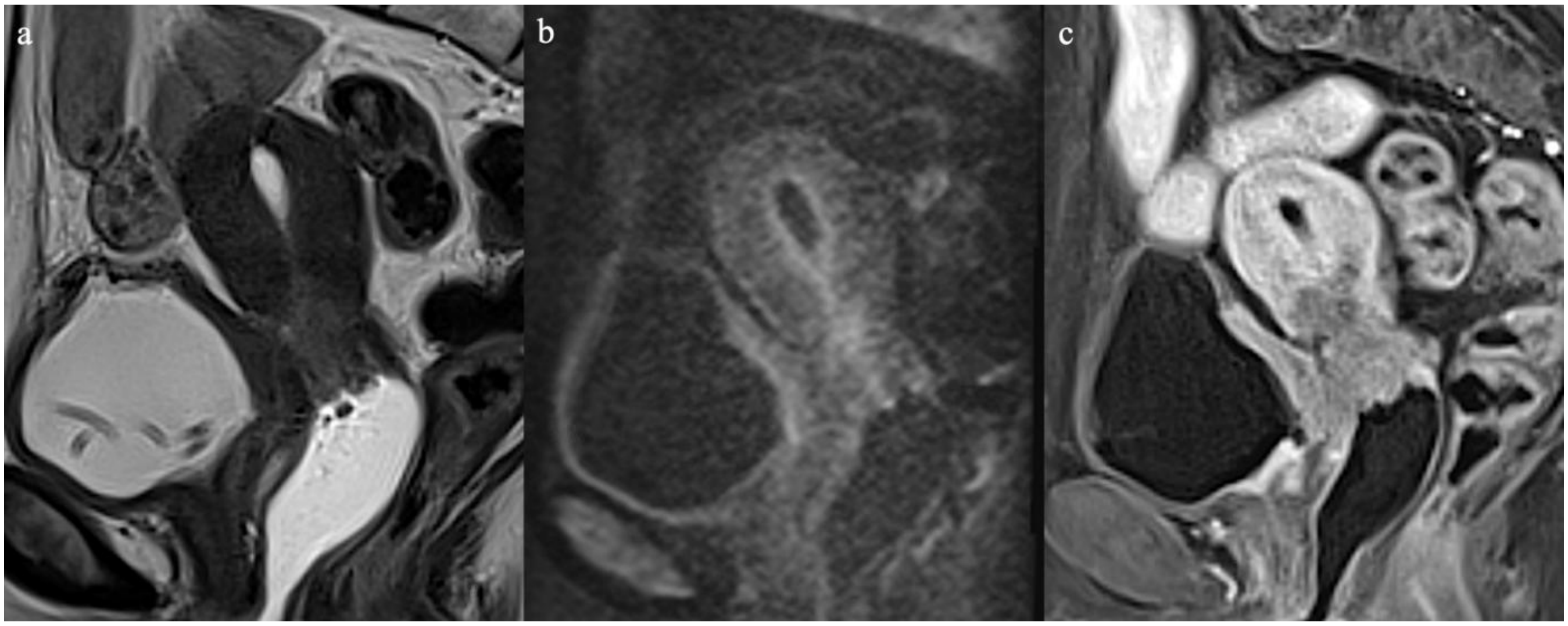

Figure S16: Cervical lipoleiomyoma.

a) sagittal T2-weighted sequence; b) axial T2-weighted sequence; c) axial in-phase T1-weighted image from a Dixon sequence; d) axial out-of-phase T1-weighted image from a Dixon sequence. MRI shows a well-defined cervical lesion (*) with relatively high T2 signal intensity. The Dixon sequence demonstrates signal loss on the out-of-phase image. Pathological examination confirmed a lipoleiomyoma.

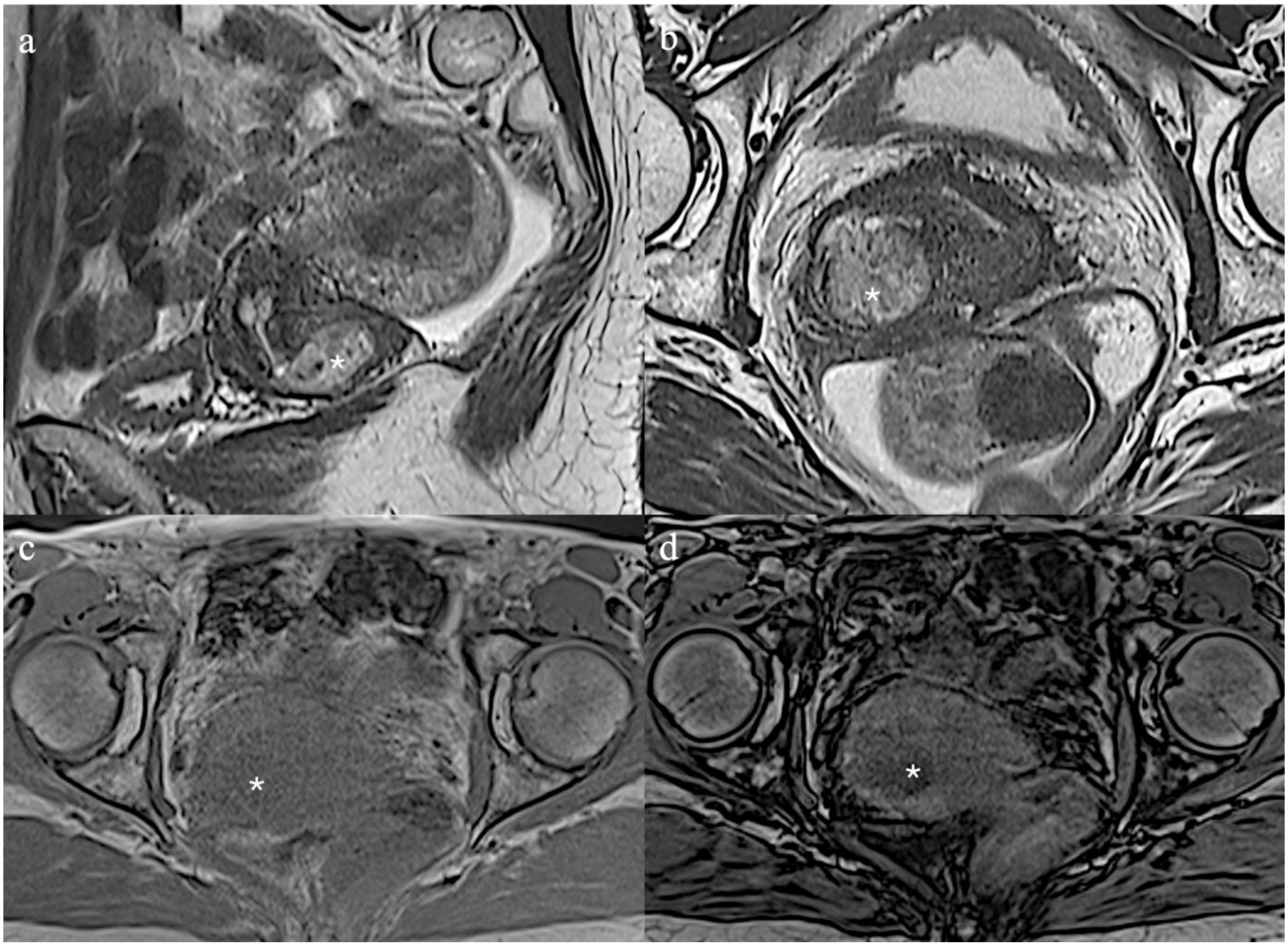

Figure S17: Cervical actinomycosis

a) sagittal T2-weighted sequence; b) sagittal post-contrast fat-saturated T1-weighted sequence; c) axial DWI sequence; d) ADC map. MRI shows a circumferential endocervical lesion (*) with relatively high T2 signal intensity, low enhancement, high signal on diffusion-weighted imaging, and low ADC values consistent with diffusion restriction. The imaging findings were considered suspicious for cervical cancer, and histopathological correlation was recommended. Actinomycosis was subsequently confirmed on tissue sampling.

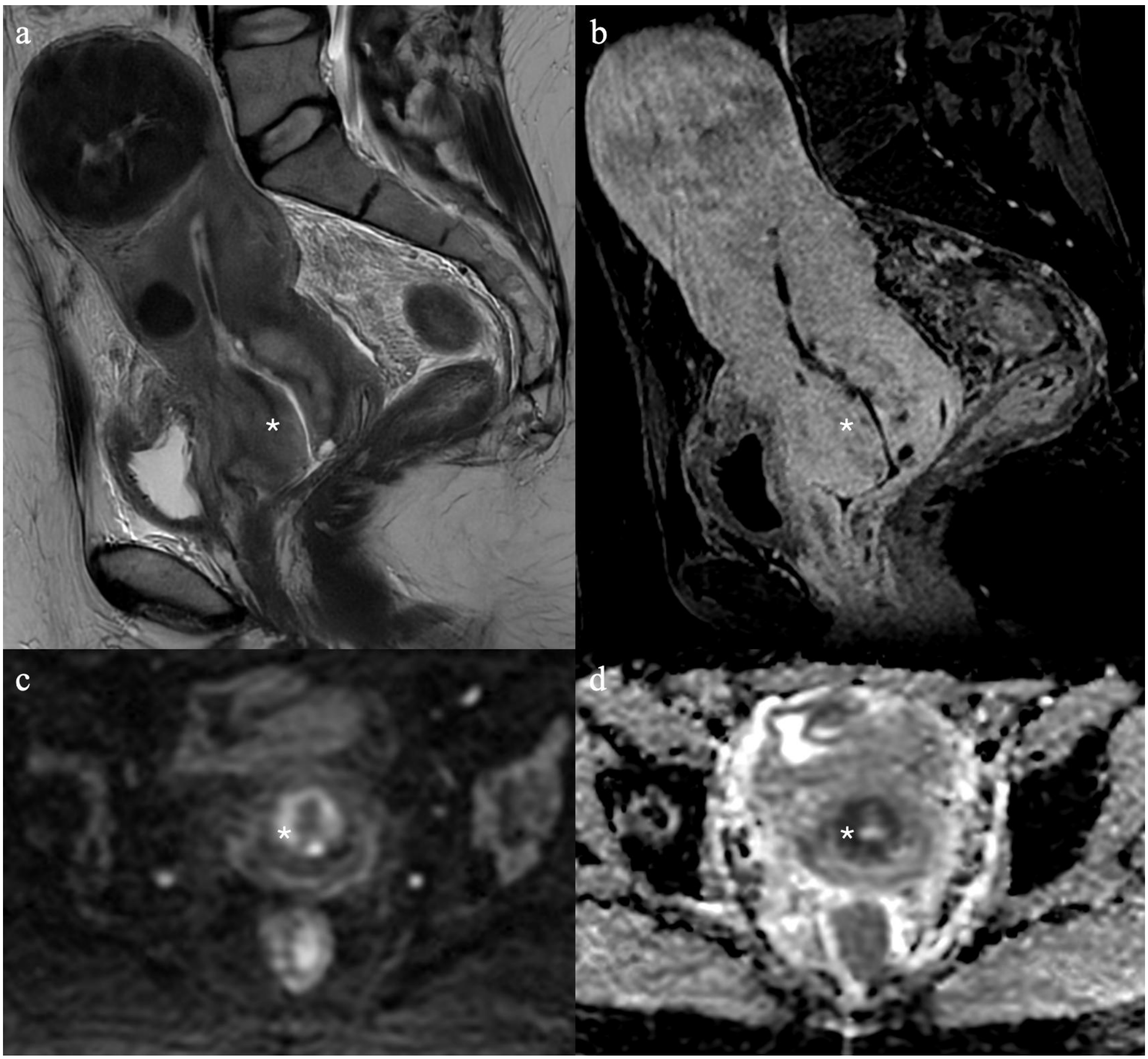

Figure S18: Typical O-RADS MR 2 solid ovarian tumor:

a) axial T2-weighted sequence; b) DWI sequence; c) TIC from DCE MR sequence. The images show a purely solid, low T2W signal right ovarian lesion (*), with a low diffusion signal. TIC from DCE MR sequence shows an intermediate-risk time intensity curve (yellow curve). The lesion was classified as O-RADS MRI score 2, corresponding to a Brenner tumor.

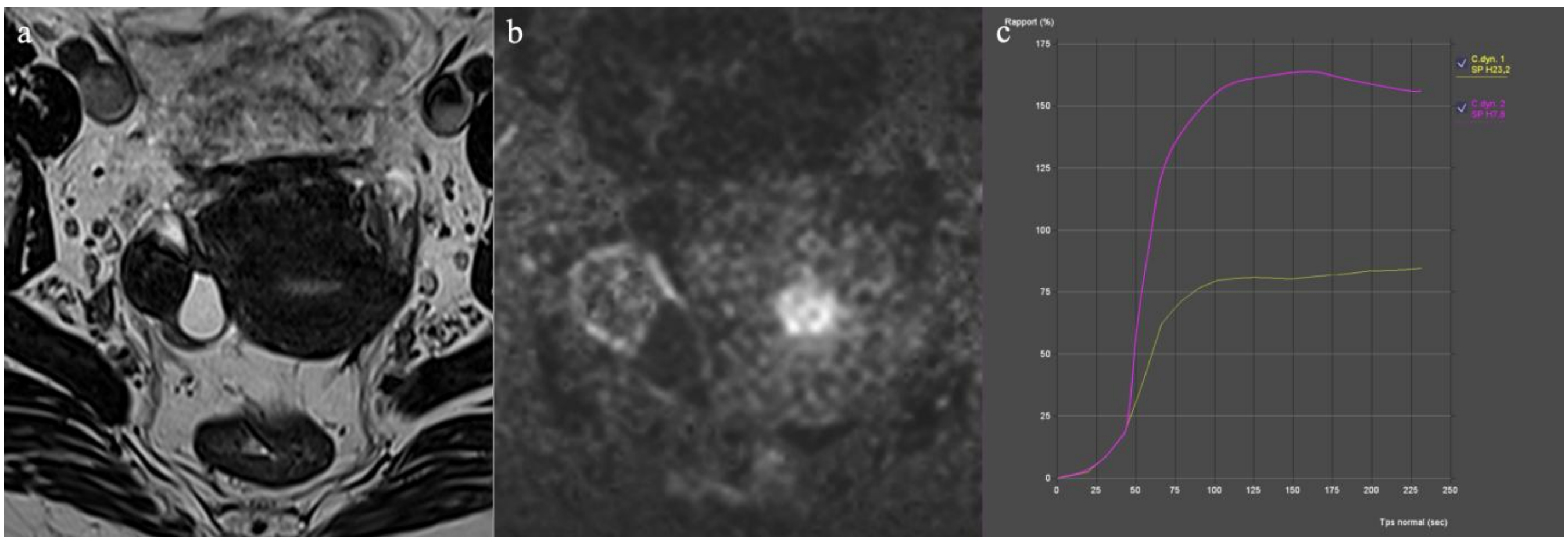

Figure S19: Mature teratoma, atypical presentations.

a) axial T2-weighted sequence; b) axial T1-weighted sequence; c) axial water-only image from a T1-weighted Dixon sequence. The images shows a purely cystic lesion with minimal fat content, consistent with a mature teratoma with poor fat content. This type of teratoma may be missed on CT and ultrasonography. Particular attention should be paid to lesions with subtle T1 signal to correctly classify them as O-RADS MRI score 2, based on the presence of diffusely distributed fat content with signal drop after fat saturation.

d) axial T1-weighted sequence; e) axial water-only image from a T1-weighted Dixon sequence; f) TIC from DCE MR sequence. Image is showing a mature teratoma with an enhancing solid component (Rokitansky protuberance). TIC from DCE MR sequence analysis of the Rokitansky shows a high-risk time intensity curve (blue curve). In the presence of a typical mature teratoma, TIC should not be performed. The lesion is classified as O-RADS MRI score 2 despite the curve.

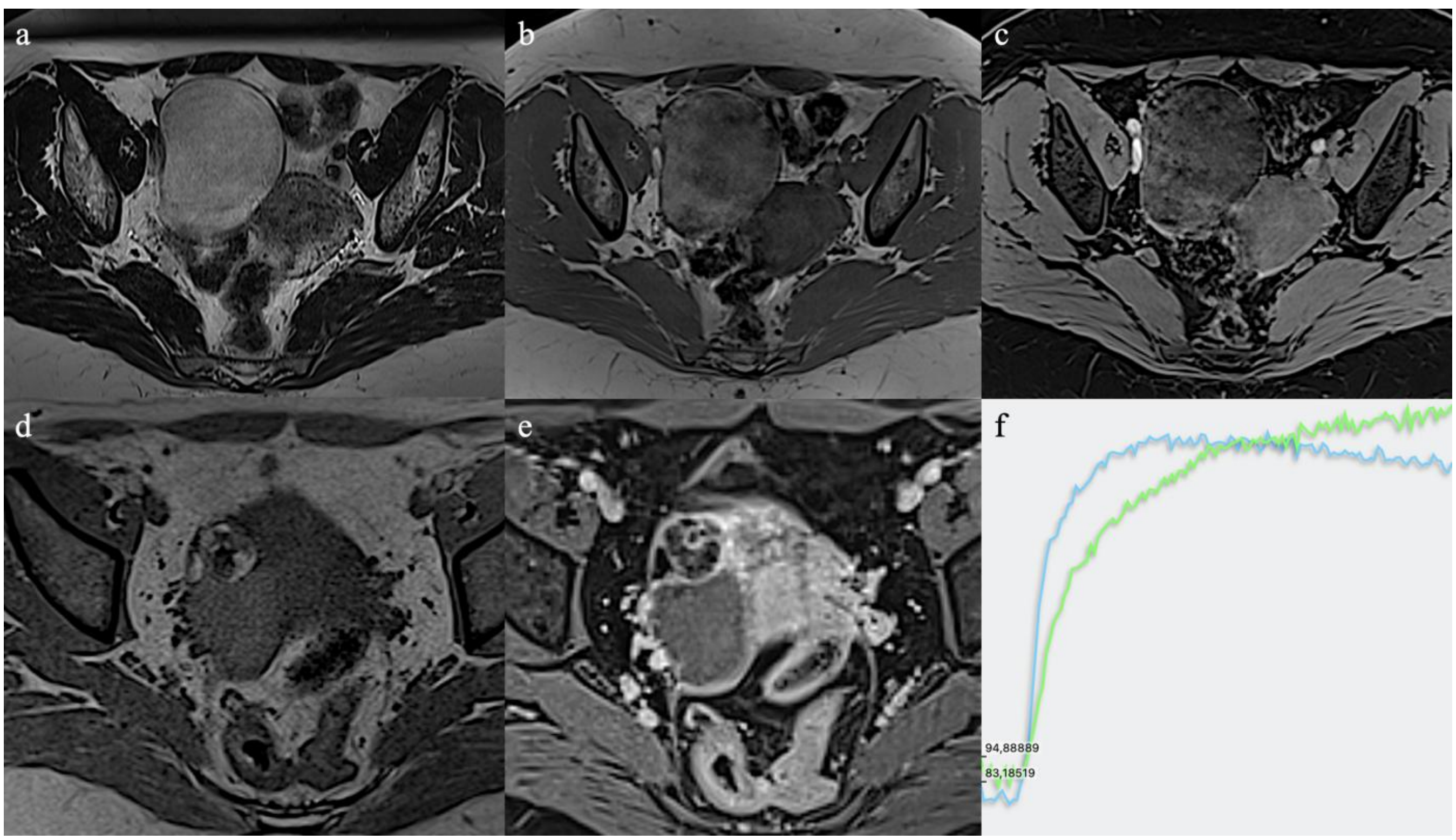

Figure S20: Necrotic ovarian tumor.

a) axial T2-weighted sequence; b) axial DWI sequence; c) axial water-only image from a T1-weighted Dixon sequence; d) axial post-contrast fat-saturated T1-weighted sequence. The images show a heterogeneous right ovarian lesion (*) with areas of high T2W signal and high diffusion signal, with spontaneous high T1W signal on T1, suggestive of hemorrhage. Post-contrast imaging shows no enhancement. O-RADS MRI was not applied because the absence of enhancement suggested a completely necrotic tumor. The patient was referred for surgery, and histopathology confirmed a mucinous carcinoma.

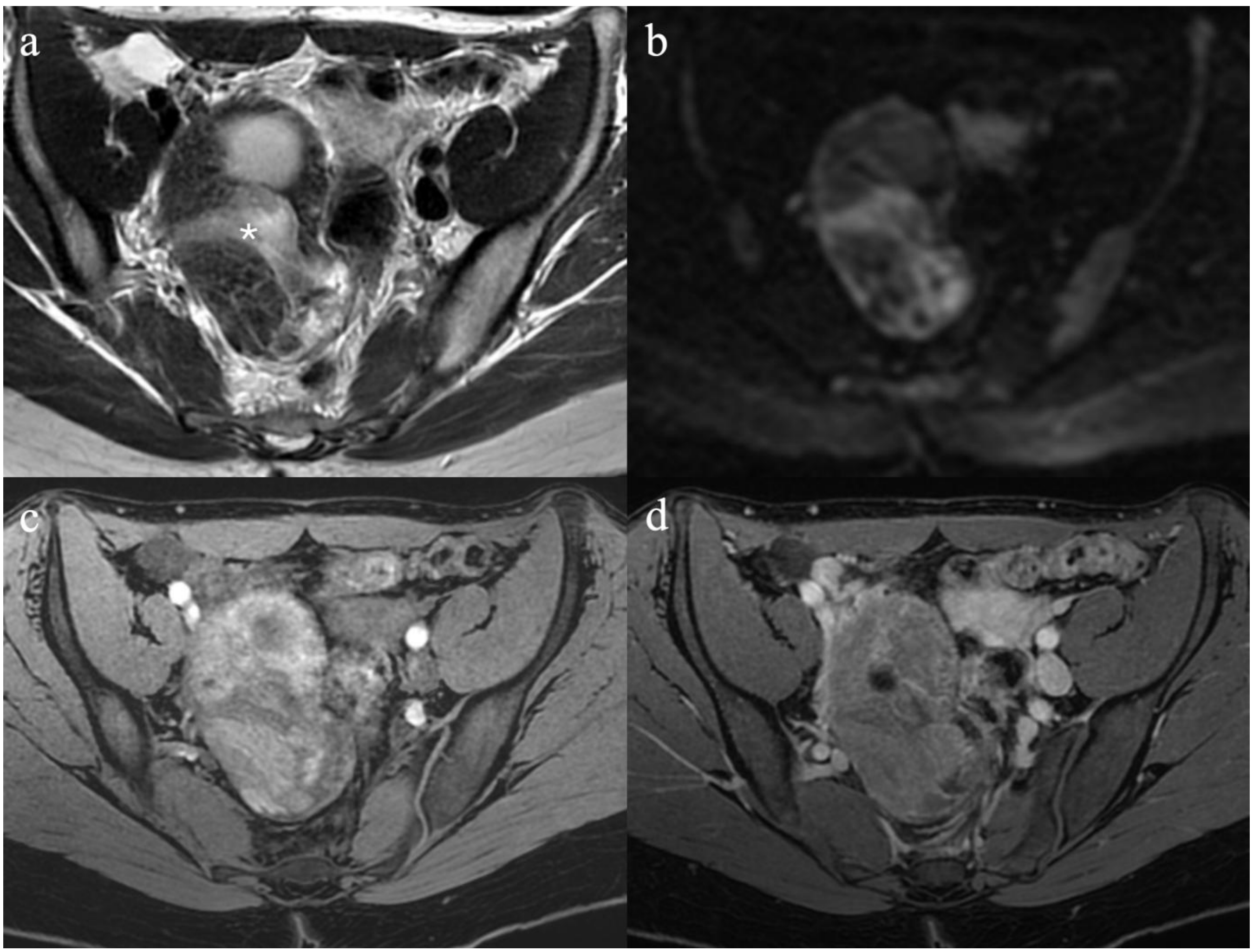

Figure S21: Cystic ovarian tumor mimickers:

a) and b) axial T2-weighted sequence. a) Shows an appendiceal mucocele mimicking an ovarian tumor but tubulated and leading to the caecum on upper slices. b) Shows a peritoneal pseudocyst showing a quadrangular cystic lesion adjacent to the left ovary.

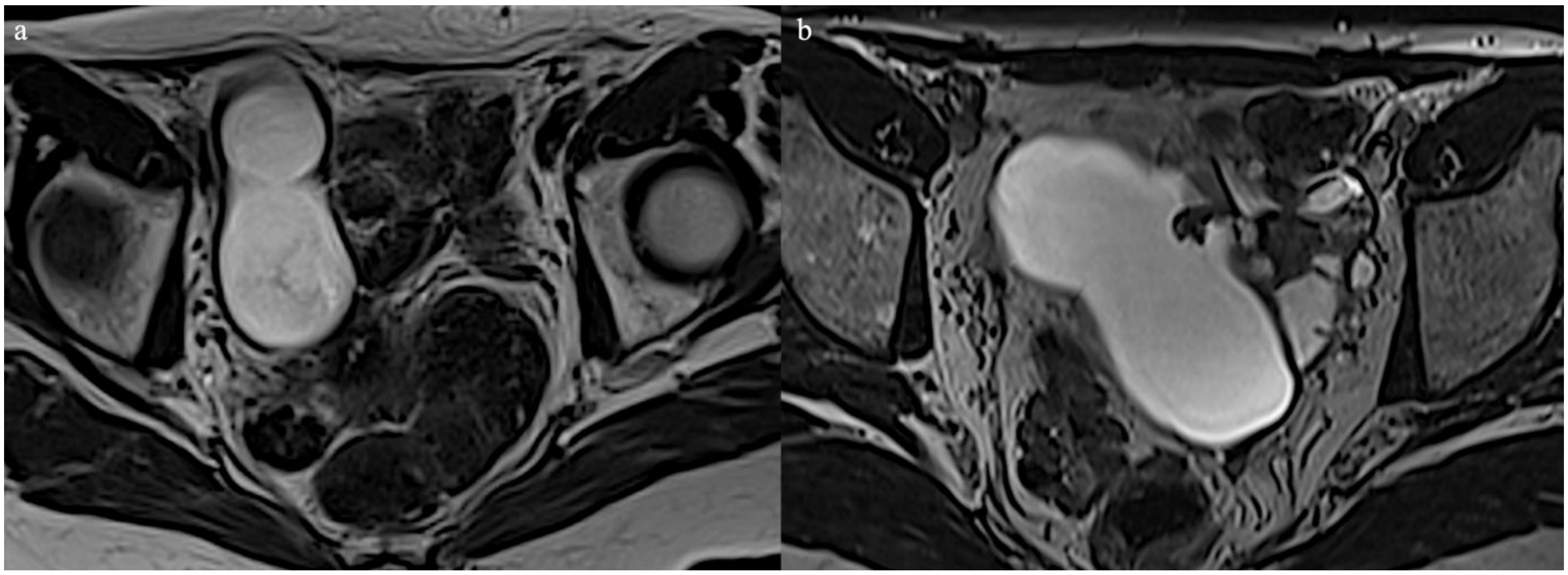

Figure S22: Solid ovarian tumor mimicker first case:

a) coronal T2-weighted sequence; b) sagittal T2-weighted sequence; c) axial T2-weighted sequence. A 45-year-old patient was referred for characterization of an adnexal mass. The images show a bilateral solid ovarian lesion (*) with peripheral low-signal rim more visible on the axial slice. After targeted questioning, the patient reported a history of medically assisted reproduction. Ovarian fibromatosis was therefore considered highly likely. Follow-up imaging confirmed lesion stability.

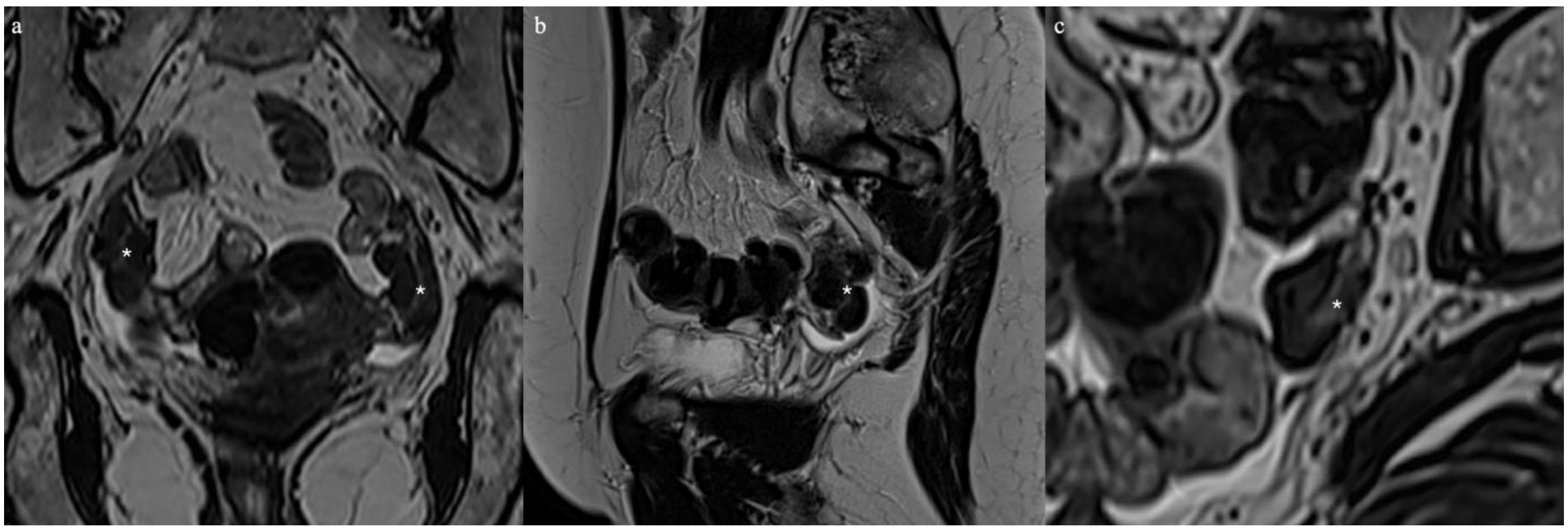

Figure S23: Solid ovarian tumor mimicker second case

a) axial T2-weighted sequence; b) sagittal T2-weighted sequence; c) axial post-contrast fat-saturated T1-weighted sequence; d) axial DWI sequence. A 45-year-old patient was referred for characterization of a very large abdominal mass. The images show a very large lesion occupying most of the abdomen with heterogeneous T2W and diffusion signal and enhancing. The ovaries were not identified. On the sagittal plane, there appears to be a possible claw-beak configuration with the posterior uterine wall. The lesion demonstrated areas of low T2 signal (*), suggesting a very large leiomyoma. The ADC map (not shown) demonstrated high values, which would give the lesion a score of 2 if myometrial (7). Given the extreme size of the mass, the patient was referred for surgical resection. Histopathology confirmed a leiomyoma with cystic and edematous degeneration.

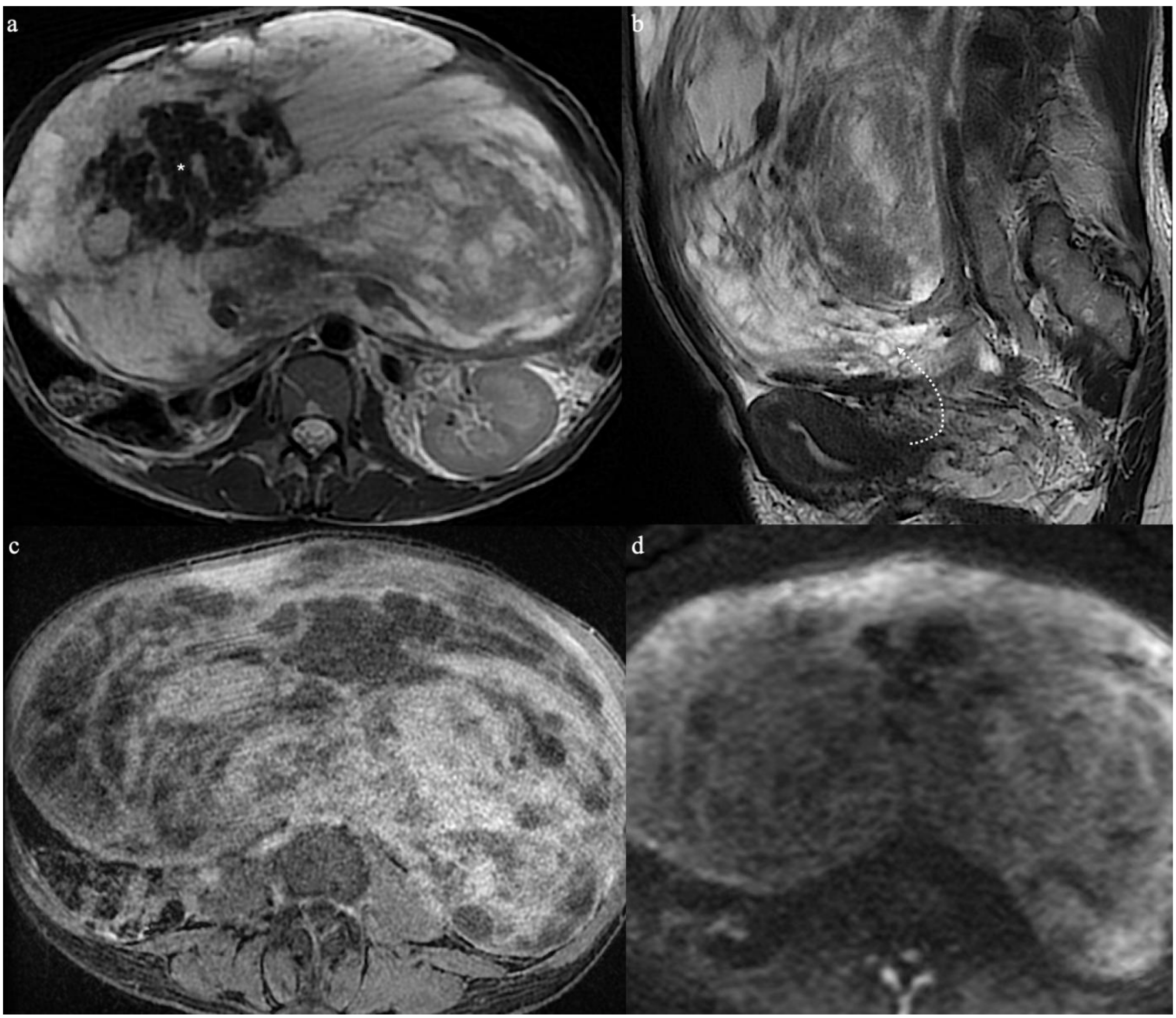

Figure S24: Disseminated peritoneal leiomyomatosis

a) and b) axial T2-weighted sequence; c) axial post-contrast fat-saturated T1-weighted sequence. A 35-year-old patient was admitted for exploration of peritoneal implants. The images show multiple round peritoneal lesions with intermediate signal intensity and homogeneous enhancement. Histopathology confirmed disseminated peritoneal leiomyomatosis.

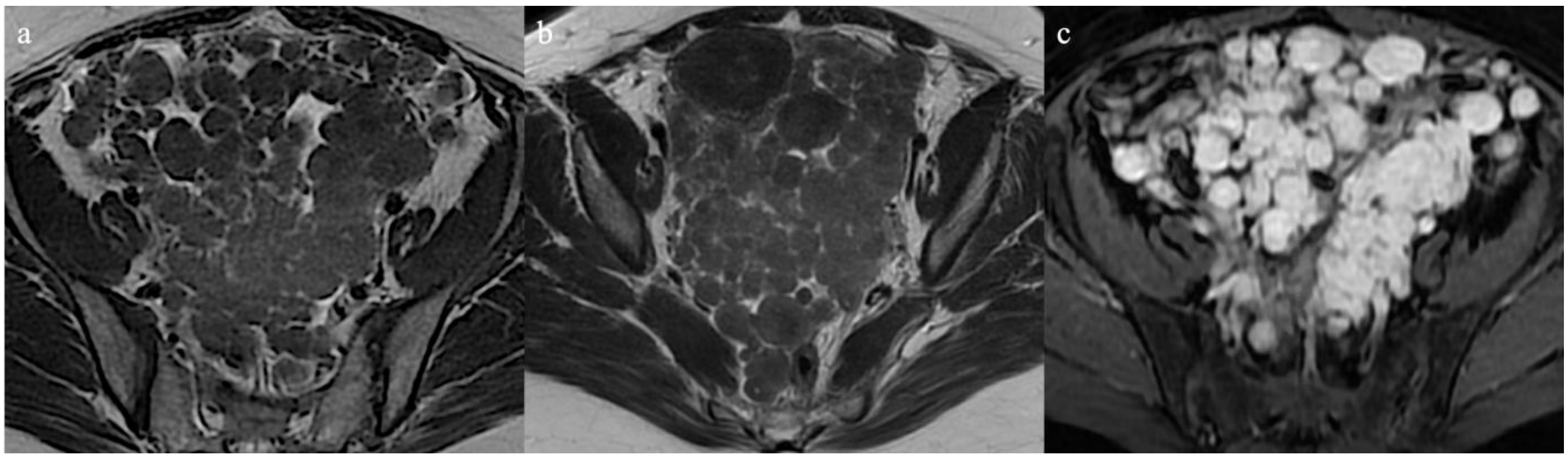

Supplement: Supplementary file 1 [file 13244_2026_2378_MOESM1_ESM.pdf]
